# Validation of a liquid biopsy assay with molecular and clinical profiling of circulating tumor DNA

**DOI:** 10.1038/s41698-021-00202-2

**Published:** 2021-07-02

**Authors:** Justin D. Finkle, Hala Boulos, Terri M. Driessen, Christine Lo, Richard A. Blidner, Ashraf Hafez, Aly A. Khan, Ariane Lozac’hmeur, Kelly E. McKinnon, Jason Perera, Wei Zhu, Afshin Dowlati, Kevin P. White, Robert Tell, Nike Beaubier

**Affiliations:** 1 Tempus Labs, Chicago, IL USA; 2 University Hospitals Seidman Cancer Center, Cleveland, OH USA

**Keywords:** Cancer genomics, Cancer genomics

## Abstract

Liquid biopsy is a valuable precision oncology tool that is increasingly used as a non-invasive approach to identify biomarkers, detect resistance mutations, monitor disease burden, and identify early recurrence. The Tempus xF liquid biopsy assay is a 105-gene, hybrid-capture, next-generation sequencing (NGS) assay that detects single-nucleotide variants, insertions/deletions, copy number variants, and chromosomal rearrangements. Here, we present extensive validation studies of the xF assay using reference standards, cell lines, and patient samples that establish high sensitivity, specificity, and accuracy in variant detection. The Tempus xF assay is highly concordant with orthogonal methods, including ddPCR, tumor tissue-based NGS assays, and another commercial plasma-based NGS assay. Using matched samples, we developed a dynamic filtering method to account for germline mutations and clonal hematopoiesis, while significantly decreasing the number of false-positive variants reported. Additionally, we calculated accurate circulating tumor fraction estimates (ctFEs) using the Off-Target Tumor Estimation Routine (OTTER) algorithm for targeted-panel sequencing. In a cohort of 1,000 randomly selected cancer patients who underwent xF testing, we found that ctFEs correlated with disease burden and clinical outcomes. These results highlight the potential of serial testing to monitor treatment efficacy and disease course, providing strong support for incorporating liquid biopsy in the management of patients with advanced disease.

## Introduction

Liquid biopsies are increasingly used as a non-invasive method for the genomic profiling of cancer. When tumor tissue is difficult or impossible to obtain, next-generation sequencing (NGS) of circulating tumor DNA (ctDNA) from blood plasma can provide valuable insights for oncologic decision making. Recently, ctDNA analysis accurately identified therapeutic biomarkers^1–5^, tumor burden^6^, resistance mechanisms^7,8^, and disease progression^9,10^.

While tissue biopsies remain the gold standard for diagnosis and biomarker identification, tumor heterogeneity can cause subclonal or emerging mutations to be overlooked, particularly in metastatic cases. Additionally, many tissue biopsies involve invasive surgical procedures that are not amenable to repeat testing or produce samples insufficient for comprehensive testing. In such cases, liquid biopsies offer several advantages, including real-time detection of emerging resistance mutations, serial testing throughout the course of treatment, and biomarker detection when tumor tissue is unavailable.

However, numerous technical limitations must be overcome to improve the clinical utility of ctDNA NGS assays. For example, many patients lack abundant ctDNA in early-stage disease and ctDNA variants may be below the limit of detection (LOD), resulting in false negatives. In addition, differentiating between germline and somatic variants in ctDNA is difficult, as is differentiating between mutations derived from clonal hematopoiesis (CH) and the solid tumor of interest. Several genes frequently mutated in CH are also important in solid tumors, including *TP53*, *GNAS*, *IDH2*, and *KRAS*^11,12^. In these cases, mutations in hematopoietic lineage cells may be mistaken for tumor-derived mutations. The ability to differentiate between germline variants, CH, and somatic tumor mutations in ctDNA will vastly improve clinical utility.

The Tempus xF assay is a 105-gene, hybrid-capture, NGS panel spanning a total of 270 kb that detects actionable oncologic targets in four variant classes: single-nucleotide variants (SNVs), insertions/deletions (indels), copy number variants (CNVs), and gene rearrangements. To establish robust clinical performance, we completed extensive validation studies that demonstrated high sensitivity and specificity, and determined a precise LOD to reduce false negatives. We show that the Tempus xF liquid biopsy detects actionable variants with high accuracy when compared to orthogonal methods, including a commercial ctDNA NGS kit, the Tempus xT NGS tissue assay, and digital droplet PCR (ddPCR).

To further evaluate the genomic landscape, we also developed a method for measuring circulating tumor fraction from targeted sequencing data. The method predicts overall tumor burden and helps distinguish germline from somatic variants in cell-free DNA (cfDNA). Many of our solid tumor clinical samples have matched normal blood samples from which buffy coat DNA is obtained. Analyzing matched samples allowed us to identify mutations resulting from CH, differentiate germline from somatic variants, and evaluate levels of tumor shedding, all of which significantly reduced false positives and false negatives.

Here, we present the analytical validation of the Tempus xF assay and characterize the potential clinical utility of estimating circulating tumor fraction to support oncologic treatment decisions.

## Results

### Tempus xF validation summary

The Tempus xF liquid biopsy oncology assay detects SNVs and indels in all 105 genes, CNVs in 6 genes, and chromosomal rearrangements in 7 genes (Supplementary Table 1). To validate the xF liquid biopsy assay, 310 samples were sequenced. Data from 11 samples were analyzed in multiple experiments for a total of 321 samples (for a breakdown of samples used in each experiment, see Supplementary Table 2). The runs generated an average of 287.7 M ± 65.6 M total reads with 143.7 M ± 32.7 M read pairs and a unique median read depth of 4579.9 ± 2305.5. The average percent of mapped reads across all runs was 99.90% ± 0.08. The median sample had a conversion efficiency of 40% (Supplementary Fig. 1).

Analytical sensitivity for all SNVs, indels, CNVs, and rearrangements targeted in the reference samples are shown in Table 1. Overall, SNVs were reliably detected at 0.25% VAF with 30 ng of input DNA (93.75% [45/48] sensitivity), indels at ≥0.5% VAF with 30 ng (95.83% [23/24] sensitivity), CNVs at ≥0.5% VAF with 10 ng (100.00% [8/8] sensitivity), and rearrangements at ≥1% VAF with 30 ng (90% [9/10] sensitivity). At ≥0.25% VAF with 30 ng of input DNA, analytical specificity was 100% for SNVs, indels, and rearrangements, and 96.2% for CNVs (Table 2).

**Table 1:** xF sensitivity.

Variant Type	DNA quantity	0.1% VAF Sensitivity, % (detected/total variants)	0.25% VAF Sensitivity, % (detected/total variants)	0.5% VAF Sensitivity, % (detected/total variants)	1% VAF Sensitivity, % (detected/total variants)	5% VAF Sensitivity, % (detected/total variants)
SNVs	10 ng	25.93 (14/54)	65.00 (39/60)	79.63 (43/54)	100.00 (39/39)	100.00 (60/60)
	30 ng	66.67 (40/60)	93.75 (45/48)	100.00 (54/54)	100.00 (51/51)	100.00 (48/48)
	50 ng	75.00 (36/48)	100 (51/51)	100.00 (60/60)	100.00 (42/42)	100.00 (60/60)
Indels	10 ng	33.33 (8/24)	46.15 (12/26)	86.36 (19/22)	100.00 (18/18)	100.00 (26/26)
	30 ng	46.15 (12/26)	75 (15/20)	95.83 (23/24)	100.00 (22/22)	100.00 (20/20)
	50 ng	45.00 (9/20)	86.36 (19/22)	96.15 (25/26)	94.44 (17/18)	100.00 (26/26)
CNVs	10 ng	0.00 (0/12)	0.00 (0/12)	100.00 (8/8)	100.00 (10/10)	100.00 (12/12)
	30 ng	0.00 (0/12)	25 (2/8)	100.00 (12/12)	100.00 (10/10)	100.00 (8/8)
	50 ng	12.50 (1/8)	20 (2/10)	100.00 (12/12)	100.00 (8/8)	100.00 (12/12)
Fusions	10 ng	0.00 (0/12)	8.33 (1/12)	37.50 (3/8)	40.00 (4/10)	100.00 (12/12)
	30 ng	16.67 (2/12)	37.50 (3/8)	75.00 (9/12)	90.00 (9/10)	100.00 (8/8)
	50 ng	0.00 (0/8)	50.00 (5/10)	66.67 (8/12)	100.00 (8/8)	100.00 (12/12)

**Table 2:** xF analytical specificity.

Variant Type	Percent	TN/(TN+FP)
SNVs	100%	264/264
Indels	100%	88/88
CNVs	96.2%	176/183
Rearrangements	100%	1848/1848

*TN* true negative, *FP* false positive.

Overall, intra-assay and inter-assay concordance between the replicates in this study was 100% for SNVs, indicating a high degree of repeatability and reproducibility. The inter-instrument concordance was 96.70% for SNVs and 100% for indels, with a combined concordance of 96.83% across instruments. Additionally, interfering substances such as genomic DNA, ethanol, and isopropanol did not cause a change in the detection of variants. Concordance between controls and samples with interfering substances was 100% among samples that passed filtering and were above the LOD.

### Accuracy of the Tempus xF assay compared to orthogonal assays

To evaluate analytical accuracy, we compared the Tempus xF assay to the Roche AVENIO ctDNA Expanded Kit. In 30 ng cfDNA samples analyzed by Tempus xF assay and AVENIO ctDNA Expanded Kit (*n* = 40), sensitivity for SNVs, indels, CNVs, and rearrangements was 94.8%, 100%, 100%, and 100%, respectively. Among the six SNVs that were not detected, five were identified but filtered out due to insufficient evidence. In 10 ng samples (*n* = 29), sensitivity for SNVs, indels, CNVs, and rearrangements was 91.9%, 100%, 80%, and 100%, respectively. Of the 7 SNVs that were not detected, 6 were identified but filtered out due to insufficient evidence (Table 3).

**Table 3:** xF accuracy compared to the Avenio ctDNA expanded kit.

Sample Size	Variant Type	Percent	Variants Called
30 ng	SNVs	94.8%	110/116
	Indels	100%	8/8
	CNVs	100%	5/5
	Rearrangements	100%	3/3
10 ng	SNVs	91.9%	79/86
	Indels	100%	3/3
	CNVs	80%	4/5
	Rearrangements	100%	1/1

**Fig. 1:**
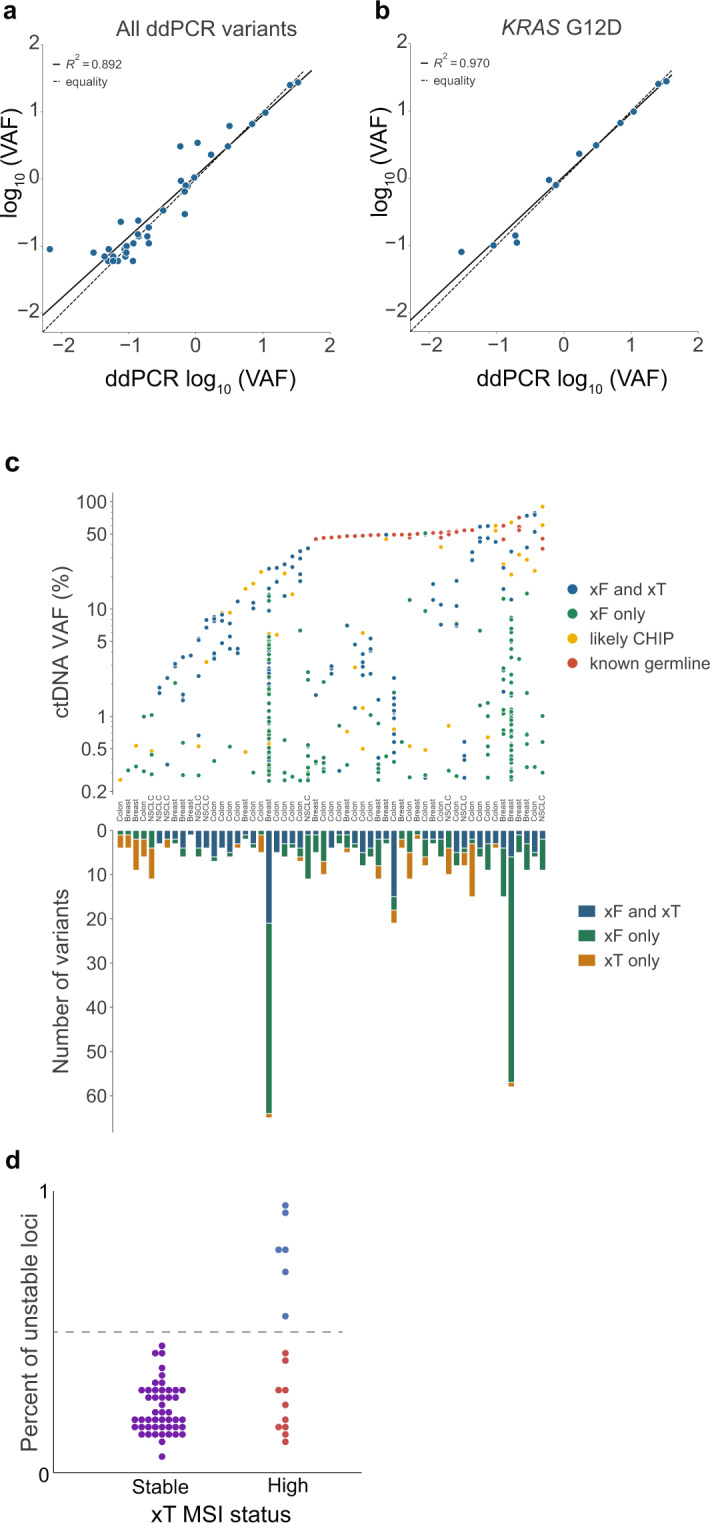
Inter-assay comparison between Tempus xF, ddPCR, and Tempus xT results. Patient samples with selected variants (*n* = 38) were analyzed by ddPCR and compared with xF variant allele fraction (VAF), resulting in high correlation overall (R^2^ = 0.892) (**a**), and in individual variants such as *KRAS* G12D (*n* = 12, R^2^ = 0.970) (**b**). **c** Results from the Tempus xF liquid biopsy and xT solid tumor assays were compared in patients who received both tests (*n* = 55) for colon, breast, and non-small cell lung cancers. The ctDNA VAFs for each variant are categorized by assay type in which they were detected and clonal hematopoiesis (CH) or germline status (top). The number of reportable variants for each individual patient are categorized by the assays in which they were detected (bottom). A total of 36 out of 55 xF samples had at least one pathogenic variant not detected in xT. **d** Among the 65 samples included in the microsatellite instability (MSI) validation cohort, 16 were deemed MSI-high and 49 microsatellite-stable. MSI was detected by xF in 6 out of 16 MSI-high patients, with 100% specificity (blue dots above dotted line).

To further validate xF assay results, patients with reported *KRAS* G12D (*n* = 12), *TERT* c.−124 (*n* = 7), *TERT* c.−146 (*n* = 5), *TP53* R273H (*n* = 7), and *TP53* R175H (*n* = 7) variants were selected for analysis by ddPCR. Then, xF NGS VAF was compared with ddPCR VAF to determine concordance. We observed 100% positive predictive value and a high correlation between ddPCR results and xF VAF (R^2^ = 0.892). The high correlation was also observed for individual variants such as *KRAS* G12D (R^2^ = 0.970) (Fig. 1a, b). Overall, these results suggest the Tempus xF assay accurately identifies hotspot mutations.

### Concordance between Tempus xF liquid biopsy and Tempus xT tissue assay

We compared analytical sensitivity and specificity in matched xT (tumor biopsy) and xF (blood biopsy) tests from 55 patients. Since xT matched samples contain both tumor tissue and buffy coat (normal comparator), we used the Tempus xT classification strategy to determine germline variants and exclude them from the analysis. After removing intronic, synonymous, benign, and likely benign variants, as well as variants below the LOD of both assays, we identified 145 concordant SNVs, 20 concordant indels, and 11 concordant CNVs. We also identified multiple discordant results, including 66 SNVs, 11 indels, and 8 CNVs that were reported in xT but not xF. Conversely, we observed 209 SNVs, 14 indels, and 7 CNVs that were reported in xF but not xT. Using the Tempus internal Bayesian dynamic filtering methodology, however, reduced this discordance by 11.45% (Supplementary Table 3). The overall sensitivity of xF relative to xT was 68.18% for SNVs and indels, and 57.89% for CNVs (Supplementary Table 4). However, when the analysis was limited to clinically actionable targets, we identified 107 concordant variants and 37 discordant variants for a final overall sensitivity of 74.31%. While CNVs are generally detected with high sensitivity by solid tumor assays, distinguishing true CNVs from noise in cfDNA is more difficult due to the relatively lower tumor fraction. Nevertheless, these values are similar to previously reported comparisons between liquid biopsy and solid tumor sequencing^13^.

Using a corresponding xT assay result, which includes germline sequencing data from buffy coat, the xF assay can also distinguish between germline and CH-derived variants. We compared the classification of reportable variants between samples with matched xF and xT testing. Variants were considered CH-derived if found in both the plasma and the xT normal sample, but not at levels consistent with germline variation (Fig. 1c). The percent ctDNA VAF (top) and number of reportable variants detected (bottom) for each individual patient were categorized by assay type and CH or germline status. Overall, we observed high concordance between the xF and xT assays (Fig. 1c and Supplementary Table 4). As expected, variants detected at higher VAFs by xF were generally detected as germline variants by xT or are likely CH variants. Notably, two samples had a large number of CH variants only detected in xF, many of which were at low VAFs. These samples were subsequently found to have very high tumor mutational burdens (TMBs) in their corresponding xT analyses. Taken together, the large number of xF variants at low VAFs and high TMBs suggest that these tumors were highly heterogeneous, and some variants are more easily detected in blood.

Lastly, the xF assay was used to assess microsatellite instability (MSI) status in samples previously classified by the xT solid tumor test or immunohistochemistry. Among the 65 samples included in the MSI validation cohort, 16 were deemed MSI-high and 49 microsatellite-stable. The xF assay reported MSI-high status in 37.5% (6/16) of orthogonally confirmed MSI-high patients at 100% (6/6) positive predictive value. Furthermore, the xF assay did not report MSI-high status for any of the 49 confirmed MSS patients (Fig. 1d). Overall, our comparison between the xT and xF assays demonstrate the strengths of the xF assay and the added value of using multiple assays to detect genomic drivers of cancer.

### OTTER, a method for estimating tumor fraction

**Fig. 2:**
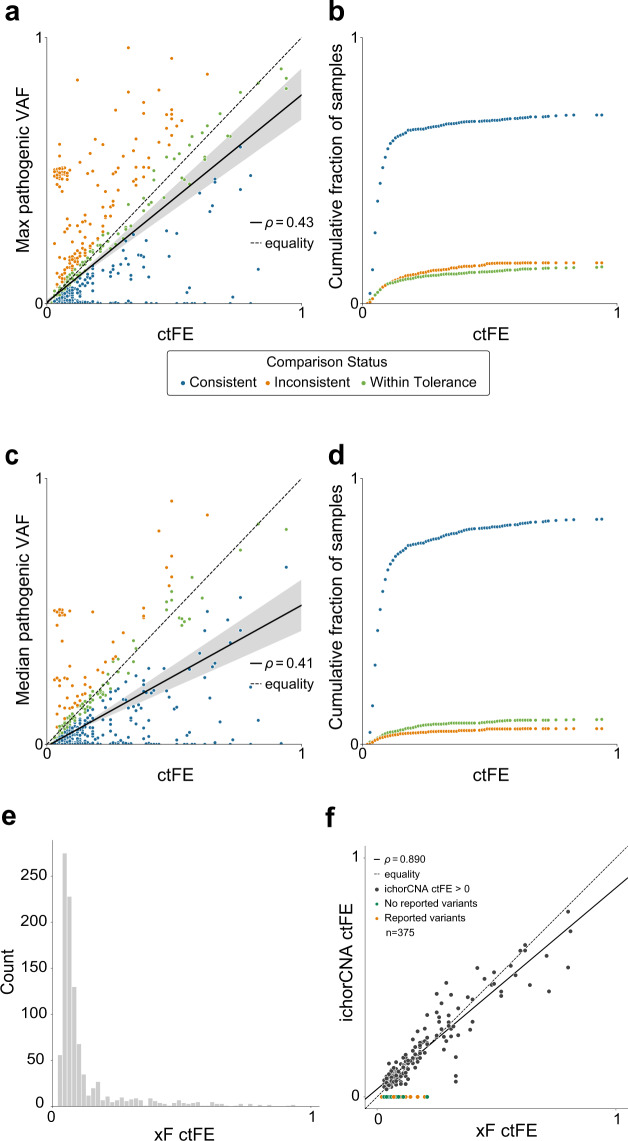
Circulating tumor fraction estimate (ctFE) and variant allele fraction (VAF). ctFE of xF-sequenced patients (*n* = 1000) shows a modest correlation with max pathogenic (**a**) and median (**c**) VAF (ρ = 0.43 and 0.41, respectively) even after removing likely germline variants and variants that fall within an amplified region of the genome. A variant can be detected at or below the tumor fraction of the sample, so if the detected VAF was less than the OTTER ctFE it was considered consistent (blue). If the maximum or median VAF was within ±20% of the relative OTTER ctFE, or an absolute 0.02 of the fraction, then the sample was considered within tolerance (green). If the detected VAF was greater than the OTTER ctFE it was considered inconsistent (orange). **b**, **d** Cumulative fraction of samples with consistent VAF and OTTER estimates of tumor fraction as the ctFE increases. Overall, 84.7% of samples had OTTER ctFEs within tolerance or consistent with the maximal VAF detected in the sample (**b**). Overall, 94.1% of samples had OTTER ctFEs within tolerance or consistent with the medial VAF detected in the sample (**d**). **e** The distribution of ctFE across the cohort (median ctFE = 0.07, mean ctFE = 0.12, SD = 0.15). **f** In samples that also underwent low-pass whole-genome sequencing (LPWGS, *n* = 375), ichorCNA detected tumor fraction in just 165 samples (black). Among those samples, there was a strong correlation between LPWGS-predicted tumor fraction and OTTER ctFE (ρ = 0.890). In the majority of samples (60%, orange) with no ctFE detected by IchorCNA, we also detected a variant using the xF assay, indicating that there was detectable tumor DNA and the ichorCNA estimate was a false negative.

Accurate measures of tumor fraction improve the understanding of variants identified through liquid biopsy testing. We developed a method, Off-Target Tumor Estimation Routine (OTTER), for determining a more accurate circulating tumor fraction estimate (ctFE). We compared OTTER ctFEs with VAFs from the xF 1000 cohort and found xF ctFE correlates with max pathogenic VAF (Fig. 2a, b) and median VAF (Fig. 2c, d) after removing germline variants and amplified regions. These results show only a modest relationship between detected VAFs and OTTER ctFEs, which we believe is primarily because VAF values provide a poor estimate of the circulating fraction. While the absolute correlation value is low, the number of samples with consistent VAF values and ctFEs is quite high (Fig. 2c, d). For the xF ctFEs to be consistent with the VAF values, we expect them to be greater than or equal to the maximal/medial somatic VAF that is not on an amplified region in a sample. Overall, after removing germline variants and variants on amplified regions, 94.1% of median VAFs were less than or equal to the corresponding xF ctFEs. The distribution of xF ctFEs for the xF 1000 cohort is shown in Fig. 2e. Overall, the median ctFE was 0.07 with a mean ctFE of 0.12.

In addition to VAF, low-pass whole-genome sequencing (LPWGS) is increasingly utilized to estimate tumor fractions and is thought to be a more accurate measure than VAF^14,15^. We compared LPWGS ichorCNA-predicted circulating tumor fraction to the OTTER ctFE in matched patient samples (*n* = 375) and found a strong correlation between methods (ρ = 0.890, *P* = 1.45e−57, Fig. 2f). This correlation indicates that OTTER ctFEs are highly concordant with estimates using LPWGS. However, OTTER exhibits a distinct advantage by calculating ctFE directly from the targeted-panel sequencing without requiring additional LPWGS analysis.

Another advantage of OTTER compared to ichorCNA is its apparent increased sensitivity for detecting tumor fraction. For 210 samples, ichorCNA estimated a tumor fraction of zero while the OTTER ctFE was greater than zero (Fig. 2f). In the majority of these samples (60%) we also detected a variant using the xF assay, indicating that there was detectable tumor DNA and the ichorCNA estimate was a false negative. Furthermore, samples with detected variants showed the same overall trend whereby the VAF was consistent with the OTTER ctFE (Supplementary Fig. 2). Finally, when ichorCNA estimates *were* greater than zero, they were consistently lower than OTTER ctFEs (Supplementary Fig. 3). Taken together these results suggest that OTTER is able to detect ctDNA in more samples than LPWGS.

### Retrospective clinical profiling of Tempus xF 1000 cohort

**Fig. 3:**
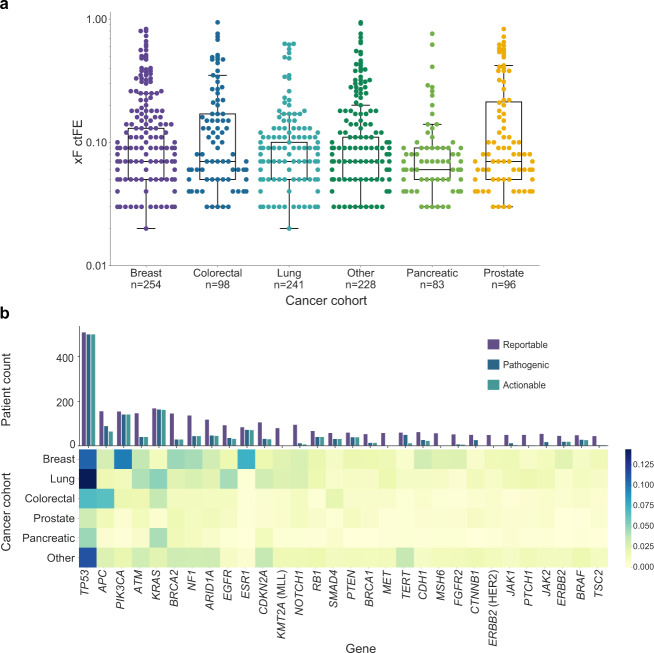
Circulating tumor fraction estimate (ctFE) and mutational landscape by cancer type. **a** Median ctFE among the most common cancer types was 0.07, with the exception of prostate (ctFE = 0.06). **b** The mutational landscape was evaluated in the xF 1000 cohort, with variants categorized as reportable, pathogenic, or actionable. Across all patients, the most commonly mutated gene was *TP53*. The heatmap was normalized within rows to depict the most prevalent variants detected for each common cancer type in the cohort (breast *n* = 254, colorectal *n* = 98, lung *n* = 241, prostate *n* = 96, and pancreatic *n* = 83).

To evaluate the clinical utility of the Tempus xF liquid biopsy, de-identified molecular and clinical data from the xF 1000 cohort were analyzed. The median ctFE predicted by OTTER was 0.07 for all cancer types, with the exception of prostate, which was 0.06 (Fig. 3a). A total of 8099 mutations were reported, of which 2732 were pathogenic and 2238 were clinically actionable (Fig. 3b). Overall, the most frequently mutated gene was *TP53* (51.1% of patients). Within cancer types, commonly mutated genes included *TP53* (41.7%)*, PIK3CA* (38.2%)*, ESR1* (29.1%)*, BRCA2* (18.5%)*, NF1* (17.3%)*, ATM* (14.6%) and *APC* (11.8%) in breast cancer, *TP53* (59.8%), *KRAS* (21.6%), *EGFR* (18.3%), and *ATM* (14.6%) in lung cancer, and *TP53* (69.4%)*, APC* (66.3%), and *KRAS (*36.7%) in colorectal cancer (Fig. 3b). These findings are consistent with the existing literature on mutated genes in each cancer type^5,10,16–19^, and indicate that the xF test accurately detects common variants of interest.

### Advanced disease is associated with higher estimated tumor fraction

**Fig. 4:**
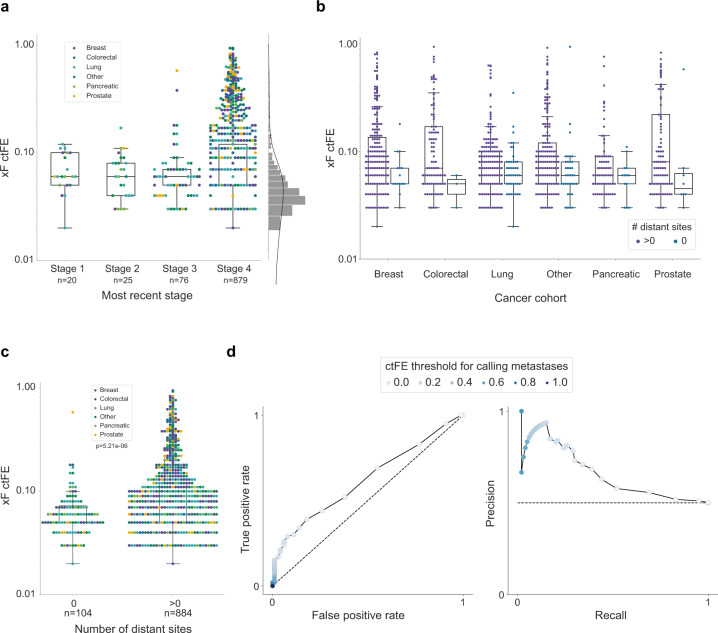
Circulating tumor fraction estimate (cfTE) according to stage and number of distant metastases. **a** Among the xF 1000 cohort, there was a significant difference in ctFE between stages (Kruskal-Wallis *P*=2.97e−5). Overall, patients with stage 4 cancer (*n* = 879, median ctFE = 0.07) had a higher ctFE than those with stages 1 (*n* = 20, median ctFE = 0.06), 2 (*n* = 25, median ctFE = 0.06), or 3 (*n* = 76, median ctFE = 0.06). ctFE increased with the number of metastatic distant sites (Mann-Whitney U test *P* = 7.57e−7) (**b**), and there was a significant difference in ctFEs between patients with no metastatic lesions (*n* = 104) and those with 1 or more distant sites affected (*n* = 884, Mann–Whitney *U* test *P* = 5.21e−6) (**c**). **d** ROC and PR curves for calling metastases at different ctFE thresholds. Samples with and without identified metastases (*n* = 100) were selected to calculate the curves with balanced classes. AUROC=0.62 and AUPR=0.64. The dashed lines show expectations when selecting samples at random.

A goal of liquid biopsy assays is to efficiently monitor treatment response and predict disease progression in patients over time. Accordingly, we investigated the association of ctFE with advanced disease states and found a significant difference in ctFEs between stages (*P* = 2.97e−5, Fig. 4a). However, since the majority of patients had advanced disease at the time of testing, additional early-stage samples are necessary to further validate these findings. We also evaluated ctFEs in patients with metastatic disease and found ctFE increases when distant sites are affected (Fig. 4b). Indeed, patients with no metastatic lesions had significantly lower ctFEs than patients with one or more distant sites (*P* = 4.77e−7, Fig. 4c, d), further highlighting the potential of ctFE for disease monitoring.

### Estimated tumor fraction correlates with response to treatment

**Fig. 5:**
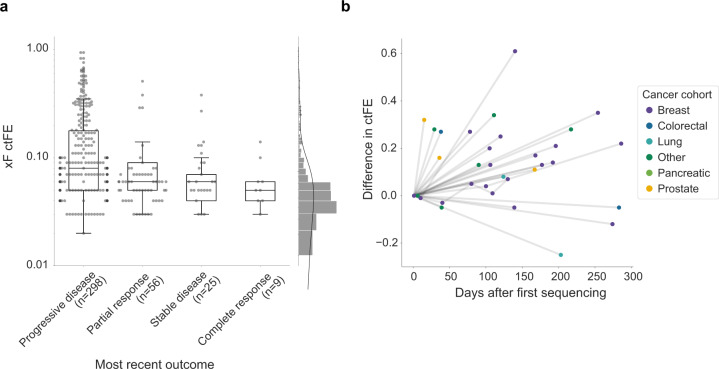
Circulating tumor fraction estimate (cfTE) and abstracted clinical outcomes in a sub-cohort of the xF 1000 (*n* = 388). **a** Patients with complete response (*n* = 9, ctFE = 0.05) exhibited lower ctFEs than those with progressive disease (*n* = 298, ctFE = 0.08), partial response (*n* = 56, ctFE = 0.06), or stable disease (*n* = 25, ctFE = 0.06). **b** ctFE was also assessed temporally among a few randomly selected patients with multiple xF tests throughout the course of treatment (*n* = 26), with most patients showing large differences in ctFEs between test dates.

To determine how ctFE changes in response to treatment, we compared ctFE with the most recent clinical response outcome. We found a significant difference (*P* = 2.34e−5 by Kruskal–Wallis) in ctFEs among patients with complete response (0.05), stable disease (0.06), partial response (0.06), and progressive disease (0.08, Fig. 5a). In addition, we found that many patients with multiple xF tests had marked differences in ctFE between test dates (Fig. 5b). While additional longitudinal studies of serial xF testing are necessary to further understand how ctFE changes in response to treatment or disease progression, these findings highlight how serial testing can benefit precision oncology.

## Discussion

Liquid biopsies are increasingly used in clinical care to identify biomarkers, detect resistance mutations, and monitor response to treatment or disease progression in real time without invasive procedures. While tissue biopsies remain the gold standard for diagnosis and identifying treatment biomarkers, liquid biopsy can add valuable insights, particularly in heterogeneous metastatic tumors with subclonal mutations. The possibility for serial testing via non-invasive liquid biopsies throughout the course of disease could prove beneficial for many patients. By monitoring emerging resistance mutations and indicators of progression, like tumor burden, clinicians are able to adjust treatment plans more nimbly.

Here, we report extensive validation studies of the Tempus xF assay that established robust technical performance, demonstrating high sensitivity and specificity for calling SNVs, indels, CNVs, and gene rearrangements. These results were highly reproducible between runs and instruments, and in the presence of interfering substances. To further establish the clinical utility of the Tempus xF assay, we evaluated the assay performance on a cohort of 1000 samples across different cancer types. We found that Tempus xF testing is clinically applicable to patients of all ages with a wide range of cancer types, mutations, disease states, and treatment histories. While it is commonly assumed that some cancer types shed little tumor DNA and are therefore less suited for liquid biopsy monitoring, we found that ctDNA was detectable at similar levels across all cancer types tested, with the exception of prostate cancer. While overall there was a correlation between metastatic lesions and circulating fraction, it is important to note that other factors such as physiology, vascular accessibility of metastatic site, and physical tumor size likely play a role in detection of ctDNA. This may also be confounded by cancer type-specific propensity for multi-organ metastatic spread.

We also present OTTER, an algorithm capable of estimating the fraction of tumor-derived circulating DNA from panel sequencing data. We demonstrated the accuracy of xF ctFEs calculated using OTTER by comparing them against the maximal and medial somatic VAFs and to estimates from LPWGS. To demonstrate the direct clinical utility of xF ctFEs, we evaluated differences in ctFEs calculated using OTTER in advanced metastatic disease. Because up to 30% of breast cancer patients and up to 55% of lung cancer patients relapse after initial treatment^20–23^, as well as a significant portion of patients in other cancer cohorts, the ability to detect metastasis and disease recurrence earlier in these patients could significantly improve outcomes. Indeed, recent retrospective and prospective studies show ctDNA measurements after completion of treatment or surgery can act as a biomarker for disease recurrence in many cancer types, including breast cancer, lung cancer, melanoma, bladder cancer, and colon cancer^24–28^. Our results are consistent with these findings, as higher ctFEs were associated with disease progression at radiographic evaluation and with an increased metastatic lesion count. Additionally, we demonstrated that ctFEs correlate with important clinical outcomes while providing a minimally invasive method to monitor patients for response to therapy, disease relapse, and disease progression.

While liquid biopsy is a promising tool for improving outcomes in precision oncology, there are challenges that must be overcome before it can replace large-panel NGS tissue genotyping. For example, in early-stage disease, when treatments have much higher success rates, many patients have low ctDNA fractions that may be below the LOD for liquid biopsies^29–32^, limiting clinical utility because of the risk of false negatives. Furthermore, while validation studies of existing liquid biopsy assays have shown high sensitivity and specificity^33–38^, few studies have corroborated results with orthogonal methods^,^ or between NGS testing platforms. Kuderer et al. compared commercially available liquid and tissue NGS platforms and found only 22% concordance in genetic alterations^39^. Other reports of liquid biopsy-based studies are limited by comparison to non-comprehensive tissue testing algorithms including Sanger sequencing, small NGS hotspot panels, PCR, and FISH. These metrics may not contain all NCCN guideline genes in their reportable range, thus suffering in comparison to a more comprehensive liquid biopsy assay^1^. Since the 105-gene Tempus xF liquid biopsy assay is a subset of the 648-gene xT tissue-based assay^40^, the concordance data presented here (74.31% for actionable variants) represents a direct comparison to a comprehensive NGS test containing the entire reportable range of the liquid biopsy assay. While this concordance is high relative to previous reports, our results nonetheless show that actionable variants would have been missed if only one of the tests were performed. The xF assay covers more genes, detects more CNVs, and includes more translocations than other commercially available methods. Overall, these benefits enhance the detection of resistance mutations. Furthermore, the method for estimating circulating tumor fraction described in the paper, OTTER, is more reliable than the median VAF values typically used in other commercially available assays. Tumor tissue profiling minimizes analytical biases driven by variable tumor shedding and the tumor percentage of the sample can be determined pre-analytically to prevent false negatives. Thus, we believe that liquid biopsies provide the greatest value to patients when used in combination with standard tissue genotyping. Furthermore, combining data from both tests enabled additional analyses to exclude germline and CH variants, significantly improving specificity.

The Tempus xF assay displays clinical utility both as a method of monitoring disease burden and as a method of detecting and responding to emerging resistance mutations. When used in the course of care, serial ctDNA monitoring can predict objective measures of progression in at-risk individuals. Due to cost and convenience of sampling, these tests can be applied at shorter time intervals than radiographic methods and allow for more timely intervention in the case of disease progression.

In summary, we present the analytical and clinical validation of the Tempus xF liquid biopsy. We show high accuracy compared to orthogonal methods, including tissue biopsy, Avenio liquid biopsy, ddPCR, and LPWGS. We also improve upon existing methodology for estimating circulating tumor fraction. Notably, we use our improved methodology and real-world clinical data to demonstrate the value and suitability of xF testing for monitoring disease progression, predicting objective measures of response, and assessing treatment outcomes. These results strongly support the Tempus xF assay’s use in routine monitoring of cancer patients with advanced disease.

## Methods

### Sample collection, storage, nucleic acid isolation, and library prep

To validate the Tempus xF liquid biopsy, 321 total specimens were analyzed. These consisted of 10 blood specimens purchased from BioIVT run in triplicate to assess inter-instrument concordance, 4 clinical samples run in triplicate to assess intra-assay concordance, 12 samples to assess inter-assay concordance, 44 residual plasma samples to assess analytical specificity, 69 clinical samples at 10 ng DNA input and 30 ng DNA input to assess analytical accuracy, and 12 clinical samples to assess interfering substances. The validation studies also included 1 cfDNA reference standard isolate (100% Multiplex I Wild Type Reference Standard HD776), and 4 cfDNA reference standards set in synthetic plasma (Horizon Discovery’s Multiplex I cfDNA Reference Standards HD812, HD813, HD814 and Horizon Discovery’s Structural Multiplex cfDNA reference standard HD786) loaded at 10 ng, 30 ng, and 50 ng of DNA input and titrated to achieve 0.1%, 0.25%, 0.5%, 1%, and 5% VAF, totaling 170 samples for evaluating xF LOD.

An additional 55 blood samples with matched tumor samples were used to compare the Tempus xF liquid biopsy and xT solid tumor tests, 65 blood samples were used to validate a microsatellite instability (MSI) classification model, and 375 blood samples were evaluated by low-pass whole-genome sequencing (LPWGS) for comparative analyses. Finally, data from an additional 1000 patient samples previously sequenced at Tempus, referred to as the xF 1000 cohort, were used for retrospective and clinical analyses. An overview of all samples included in the validation and retrospective profiling experiments is presented in Supplementary Table 5.

All blood was received in Cell-free DNA BCT^®^ blood collection tubes (Streck) within 36 h of collection and stored at RT or 4 °C until plasma separation. Plasma was prepared immediately after accessioning and stored at −80 °C until nucleic acid extraction and library prep. At this time, cfDNA was isolated from plasma using the Qiagen QIAamp MinElute ccfDNA Midi Kit (QIAGEN) according to manufacturer’s instructions. Automated library preparation was performed on a SciClone NGSx (Perkin Elmer). All cfDNA samples were normalized with molecular grade water to a maximum of 50 μL.

### xF sequencing assay

This assay uses New England BioLab’s NEBNext® Ultra™ II DNA Library Prep Kit for Illumina^®^, IDT’s xGen CS Adapters, unique molecular indices (UMI), and 96 pairs of barcodes to prepare cfDNA sequencing libraries with unique sample IDs. Each sample is ligated to a dual unique index, which enables multiplexed sequencing of up to 7 patients and 1 positive control per SP NovaSeq flow cell, 16 patients and 1 positive control per S1 NovaSeq flow cell, 34 patients and 1 positive control per S2 NovaSeq flow cell, and 84 patients and 1 positive control per S4 NovaSeq flow cell. The library preparation protocol is optimized for ≥20 ng cfDNA input to maximize mutation detection sensitivity. The final library is sequenced on an Illumina NovaSeq sequencer and analysis is performed using Tempus’ bioinformatics pipeline and analysis server.

### Bioinformatics pipeline

Adapter-trimmed FASTQ files are aligned to the 19th edition of the human reference genome build (hg19) using Burrows-Wheeler Aligner (BWA)^41^. Following alignment, reads are grouped by alignment position and UMI family, and collapsed into consensus sequences using fgbio tools (http://fulcrumgenomics.github.io/fgbio/). Bases with insufficient quality or significant disagreement among family members are transformed to N’s. Phred scores are scaled based on initial base calling estimates combined across all family members. Following single-strand consensus sequence generation, duplex consensus sequences are generated by comparing the forward- and reverse-oriented PCR products with mirrored UMI sequences. Consensus sequences are re-aligned to the human reference genome using BWA. BAM files are generated and indexed after the re-alignment.

SNV and indel variants are detected using VarDict^42^. SNVs are called down to 0.1% VAF for specified hotspot target regions and 0.25% VAF at all other base positions across the panel. Indels are called down to 0.5% VAF for variants within specific regions of interest. Any indels outside of these regions are called down to 5% VAF. All SNVs and indels are then sorted, deduplicated, normalized and annotated. Following annotation, variants are then classified as germline, somatic, or uncertain using a Bayesian model. The model is based on prior expectations informed by internal and external databases of germline and somatic variants. Uncertain variants are treated as somatic for filtering and reporting purposes. Following classification, variants are then filtered based on a set of quality metrics including coverage, VAF, strand bias, and genomic complexity. Additionally, variants are filtered with a Bayesian trinucleotide context-based model with position-level background error rates estimated from process-matched healthy controls. Known artifactual variants are also removed.

Copy number variants (CNVs) are analyzed using CNVkit^43^ plus a Tempus CNV annotation and filtering algorithm. CNVkit is used for genomic region binning, coverage calculation, bias correction, normalization to a reference pool, segmentation, and visualization. The log2 ratios between the tumor sample and a pool of process-matched healthy samples from the CNVkit output are then annotated and filtered using statistical models whereby the amplification status (amplified or not-amplified) of each gene is predicted and non-focal amplifications are removed.

Rearrangements are detected using the SpeedSeq analysis pipeline^44^. Briefly, FASTQ files are aligned to hg19 using BWA. Split reads mapped to multiple positions and read pairs mapped to discordant positions are identified and separated, then used to detect gene rearrangements by LUMPY^45^. Fusions are then filtered by the number of supporting reads.

Predicted functional effect and clinical interpretation for each variant are curated by automated software using information from both internal and external databases. The software uses a weighted-heuristic model with logic-based recommendations from the AMP/ASCO/CAP/ClinGen Somatic working group^46^ and ACMG guidelines^47^.

To detect MSI, the relative frequency and distribution are determined for any read containing repetitive sequences. To predict the probability of an unstable locus, a k-nearest neighbors model (with k = 100) is used along with normalized percent lower, mean lower, and mean log-likelihood metrics. The percentage of unstable loci is calculated from the probabilities of each sample, with >50% unstable loci considered MSI-high.

### Validation approach

To establish robust technical performance, extensive validation studies were performed. LOD was determined by assessing analytical sensitivity in reference standards with 5%, 1%, 0.5%, 0.25%, and 0.1% VAF generated from the Horizon Discovery reference set. The Horizon Discovery set includes 160 bp cfDNA fragments from human cell lines in an artificial plasma matrix to closely resemble cfDNA extracted from human plasma. VAFs of SNVs and indels, including *EGFR (ΔE746 - A750), EGFR (V769 - D770insASV), EGFR A767_V769dup, EGFR (L858R), EGFR (T790M), KRAS (G12D), NRAS (A59T), NRAS (Q61K), AKT1 E17K, PIK3CA (E545K)*, and *GNA11 Q209L* were measured in reference samples by the xF assay. The reference samples were also evaluated for CNVs and rearrangements, including *CCDC6/RET, SLC34A2/ROS1*, *MET*, *MYC*, and *MYCN*. Each measurement was run with a minimum of three replicates at 10 ng, 30 ng, and 50 ng of DNA. Sensitivity was calculated by the number of detected variants divided by the total number of variants present in the reference samples. Samples with an on-target rate <30% were excluded from this analysis and *MET* (4.5 copies) was not included in the CNV sensitivity calculation. Sensitivity >90% was considered reliable detection.

Analytical specificity was determined using 44 normal samples titrated at 1%, 2.5%, or 5% from a wild-type cfDNA reference standard (HD776) with a list of SNVs and indels that are known to be absent and were considered true-negative variants for the specificity calculation. False-positive variants were those identified as pathogenic or likely pathogenic after subtracting the background variants from HD776 from all reported variants in the 44 samples. Since there is no existing standard with CNVs and rearrangements that are known to be absent, the number of reportable CNVs and rearrangements that were not identified in HD776 was used as a true-negative count for CNV and rearrangement specificity. Specificity was calculated by the number of known true-negative variants divided by the number of true-negative variants plus false-positive variants identified by the xF assay.

To assess inter-instrument concordance between the sequencing instruments, 10 patient libraries were sequenced on each instrument (3 NovaSeqs). Variants observed below the lower limit of detection (LLOD) (0.25% for SNVs and 0.50% for indels) were excluded from concordance analysis.

To evaluate reproducibility, inter-assay variant concordance of 4 clinical samples with variants near the target sensitivity of the assay were analyzed in three separate runs. The three separate runs were prepared with different barcodes and run on separate days by three different technologists. The four clinical samples used for inter-assay concordance were previously used in inter-instrument concordance. Variant concordance was evaluated for those variants that were above the LOD.

To evaluate repeatability, the same four clinical samples used in the inter-assay concordance were analyzed in triplicate within the same run using different barcodes. These samples contained variants near the assay target sensitivity. Variant concordance was evaluated for those detected above the LOD.

To establish analytical accuracy, the xF results from 40 validation samples with 30 ng DNA input and 29 validation samples with 10 ng DNA input were compared to the results of an orthogonal reference method (Roche’s AVENIO ctDNA Expanded Kit, Cat# 8061076001). Analytical accuracy was calculated by the number of detected variants divided by the total number of variants present in the sample. Variants that were off-target or below the LLOD (0.25% for SNVs and 0.5% for indels) were excluded from the analysis.

The effect of ethanol, isopropanol, and genomic DNA interference on sequencing was evaluated using three clinical samples from the analytical accuracy experiment. The clinical samples were normalized to 45 µL with 5 µL of the interfering substance spiked in prior to sequencing. Concordance between control samples and those containing interfering substances was evaluated for samples that passed filtering criteria and for variants above the LOD.

### Digital droplet polymerase chain reaction (ddPCR)

Five variants were validated on the ddPCR platform: *KRAS* G12D (Integrated DNA Technologies, IDT, published sequences), *TERT* promoter mutation c.−124C > T (C228T), *TERT* promoter mutation c.−146C > T (C250T) (Thermo Fisher Scientific), *TP53* p.R273H, and *TP53* p.R175H (Thermo Fisher Scientific). Each amplification reaction was performed in 25 μL and contained 1X Genotyping Master Mix (Thermo Fisher Scientific), 1X droplet stabilizer (RainDance), 1X of primer/probe mix for *TERT* and *TP53* (for *KRAS*: 800 nM of each primer and 500 nM of each probe) plus template. To improve the LLOD, 4-cycle amplification was performed prior to droplet generation. Amplification for *KRAS* was performed using the following cycling conditions: 1 cycle of 95 °C (0.6 °C/s ramp) for 10 min, 4 cycles of 95 °C (0.6 °C/s ramp) for 15 sec and 60 °C for 2 min, followed by 1 cycle of 98 °C (0.6 °C/s ramp) for 10 min. Cycling conditions for the *TP53* variants were the same as those for *KRAS*, except the annealing and extension occured at 55 °C for 2 min. Amplification for *TERT* followed Thermo Fisher’s recommendation: 1 cycle of 96 °C (1.6 °C/s ramp) for 10 min, 4 cycles of 98 °C (1.6 °C/s ramp) for 30 sec and 55 °C for 2 min, followed by 1 cycle of 55 °C (1.6 °C/s ramp) for 2 min. Droplets were then generated on the RainDance Source, and amplification was performed following the above cycling conditions with 45 cycles for both *KRAS* and *TP53*, and 54 for *TERT*. Droplets were placed on the RainDance Sense droplet reader, and data were acquired and analyzed using RainDrop Analyst II v1.1.0.

### Concordance between Tempus xF and xT assays

Matched xF (liquid biopsy) and xT (solid tumor) sample pairs (*n* = 55) from colon (*n* = 28), breast (*n* = 18), and lung (*n* = 9) cancers were used to calculate analytical sensitivity and specificity. Solid tumor and matched normal samples obtained from peripheral blood buffy coat were analyzed with the Tempus xT assay, and corresponding blood plasma samples were analyzed with the Tempus xF liquid biopsy assay. Since the 105-gene Tempus xF liquid biopsy assay is a subset of the 648-gene xT tissue-based assay^40^, only variants in the reportable range of both the xT and xF panels were included in these analyses. Germline, intronic, and synonymous variants identified in xT and xF were excluded from analysis with the exception of intronic splice variants. To determine analytical sensitivity, the number of variants identified in both xF and xT (true positives) was divided by the sum of true positives and those identified only in xT. To determine analytical specificity, the number of positions reported in neither xF nor xT (true negatives) was divided by the sum of true negatives and variants only identified in xF.

To improve xF variant calling, we developed a strategy that dynamically calculates local sequence errors using Bayes Theorem and the likelihood ratio test. The dynamic threshold is calculated using a sample-specific error rate, the error rate from healthy control samples, and from our internal cohort of solid tumor samples. This method was tested on 55 matched xF/xT samples, with variants detected in the xT solid tumor assay as the source of truth. Using sensitivity thresholds defined by the LOD analysis, fixed post-test odds (equal to the P(post-test) / [1 - P(post-test)]) and pre-test odds (calculated using the same equation but with historical data from the xT assay), we developed the following formula:

1 $$specificity = 1 - pre\!-\! test\,odds \ast sensitivity/post\!-\! test\,odds$$

The specificity was input to a beta-binomial function and yielded the minimum number of alternate alleles to call a variant at a given depth. The pre-test odds metric was specific to individual cancer cohorts and individual genes, allowing for cancer-specific pre-test odds to be applied to individual exons.

### Estimation of circulating tumor fraction

Existing methods for calculating tumor purity either require a separate sequencing experiment^14^ or a large number of targets^48^ for accurate estimation. Therefore, circulating tumor fraction estimates (ctFEs) were determined using an Off-Target Tumor Estimation Routine (OTTER) from both on- and off-target reads distributed across the human reference genome. OTTER is distinct from other methods in its generation of a ctFE from a single targeted-panel sequencing experiment.

As described above, CNVkit was run on each sample and segments were assigned via circular binary segmentation (CBS)^49^. Segments were then fit to integer copy states via an expectation-maximization algorithm using the sum of squared errors of the segment log2 ratios (normalized to genomic interval size) to expected ratios given a putative copy state and tumor purity. Following these methods, ctFEs were generated for the xF 1000 cohort.

OTTER begins with the output of CNVkit that defines a set of copy ratio segments, S

2 $$S = \left\{ {CR_1,CR_2, \ldots ,CR_n} \right\}$$

where each segment, *CR*_*i*_ is defined as

3 $$CR_i = {\it{log}}_2\left( {\frac{{normalized\,sample\,coverage}}{{normalized\,pool\,coverage}}} \right)$$

and can vary in length depending on the number of bins in the segment assigned by the CBS algorithm in CNVkit. The set of segments are filtered based on two criteria. A segment is removed if it is on a contig that is historically difficult to sequence, such as the sex chromosomes X and Y. A segment may also be removed if it is too short. In this case we removed segments with fewer than 100 bins.

Next, based on the user parameters for possible tumor fractions (*T)* and copy states (*C)* to check, OTTER calculates a *t* x *c* matrix of expected *log2* copy ratios, *E*. In this implementation we used tumor fractions ranging from 1 to 99% in increments of 1%

4 $$T = \{ 0.01,0.02, \ldots ,0.99\}$$

And copy states

5 $$C = \{ 0,1,2,3,4\}$$

however, different sets of *T* and *C* can be specified if desired. Each expected *log2* copy ratio *E*_*t,c*_ for tumor fraction *t* and copy state *c* can therefore be calculated as

6 $$E_{t,c} = log_2\left( {\frac{{2(1 - t) + t \times c}}{2}} \right)$$

For example, the expected *log2* copy ratio for a segment with 4 copies of the genome at a tumor fraction of 50% would be

7 $$E_{0.5,4} = {\it{log}}_2\left( {\frac{{2(1 - 0.5) + 0.5 \times 4}}{2}} \right) = {\it{log}}_2\left( {\frac{3}{2}} \right) = 0.58$$

Using this expectation matrix the distance of a calculated copy ratio segment *i* to each copy state in *C*, for a given tumor fraction *t* can be calculated as

8 $$d_{i,t} = \left( {CR_i - E_{t,c}} \right)^2\forall c \in C$$

Therefore, the closest copy state, *c*_*i,t*_, for the segment at the given tumor fraction, *t*, is determined as

9 $$c_{i,t} = argmin_c(d_{i,t})$$

the weighted error associated with that segment is

10 $$\varepsilon _{i,t} = min(d_{i,t}) \times l_i$$

where *l*_*i*_ is number of bins on the segment. Finally, the loss at a tumor purity *t* is calculated as

11 $$loss_t = \mathop {\sum}\nolimits_i {\varepsilon _{i,t}}$$

and the final tumor purity estimated by OTTER is

12 $$\hat t = argmin_t\left( {loss_t} \right)\forall t \in T$$

### Low-pass whole-genome sequencing and analysis

To confirm the accuracy of OTTER, blood samples from 375 patients with xF testing were also sequenced using LPWGS across four flowcells. Sequencing coverage metrics for these samples were calculated using *Picard CollectWgsMetrics*. The tumor fraction and ploidy values for each sample were estimated using *ichorCNA*^14^ with a Tempus-specific panel of 47 normal samples. This approach provides tumor fraction estimates from WGS to compare against ctFEs generated from xF targeted-panel sequencing data. Reported variants from the corresponding xF analysis of each sample were used to assess the accuracy of the ctFE.

### Clinical profiling of the xF 1000 cohort

A cohort of 1000 de-identified patient health records was randomly selected from the Tempus clinicogenomic database for analysis. The cohort included non-hematologic malignancies of known cancer type and stage previously sequenced with the xF assay. All data were de-identified in accordance with the Health Insurance Portability and Accountability Act (HIPAA) using Safe Harbor guidelines. Dates used for analyses were relative to the first xF sequencing date of each patient, and year of the first sequencing date was randomly off-set. The cohort comprised 55.7% female and 44.3% male patients, with a median age of 66 years and interquartile range of 15. The cohort also spanned 24 cancer categories (Supplementary Table 2), with breast (*n* = 254), colorectal (*n* = 98), lung (*n* = 241), pancreatic (*n* = 83), and prostate (*n* = 96) being the most common. Only variants classified as pathogenic or likely pathogenic were included in the analyses. These variants were further classified as actionable if matched to diagnostic, prognostic and/or therapeutic evidence, or if considered biologically relevant. Outcomes were determined according to the most recent clinical response noted in patient records. The study protocol was submitted to the Advarra Institutional Review Board (IRB), which determined the research was exempt from IRB oversight and approved a waiver of HIPAA authorization for this study.

### Reporting summary

Further information on research design is available in the Nature Research Reporting Summary linked to this article.

## Supplementary information

Supplementary Information

Reporting Summary

## Data Availability

The data generated and analyzed during this study are described in the following data record:10.6084/m9.figshare.14672673^50^. The de-identified clinical data that support findings from the retrospective profiling study have been deposited in the Vivli repository (https://vivli.org/) in the file ‘xf_1000_De-identified_Phenotypic_Data_and_ctFE.tsv’ under the following accession: T20.01^51^. Access is restricted and interested parties must make an authorized request to the Vivli repository. Supplementary Tables 1–4 are openly available in accessible format as part of the data record^50^. Raw data from the validation experiments were generated and analyzed as part of a CAP/CLIA validation. As such, they are not publicly available but have been thoroughly reviewed by those governing authorities.

## Code availability

Requests for source code are subject to review by Tempus and can be directed to the lead author.
