# Supplementary material for: Validation of a liquid biopsy assay with molecular and clinical profiling of circulating tumor DNA
Source: NPJ Precis Oncol. 2021 Jul 2;5:63. doi: 10.1038/s41698-021-00202-2 (PMC8253837; doi:10.1038/s41698-021-00202-2)
Supplement: Supplementary file 1 — Supplementary Information [file 41698_2021_202_MOESM1_ESM.pdf]

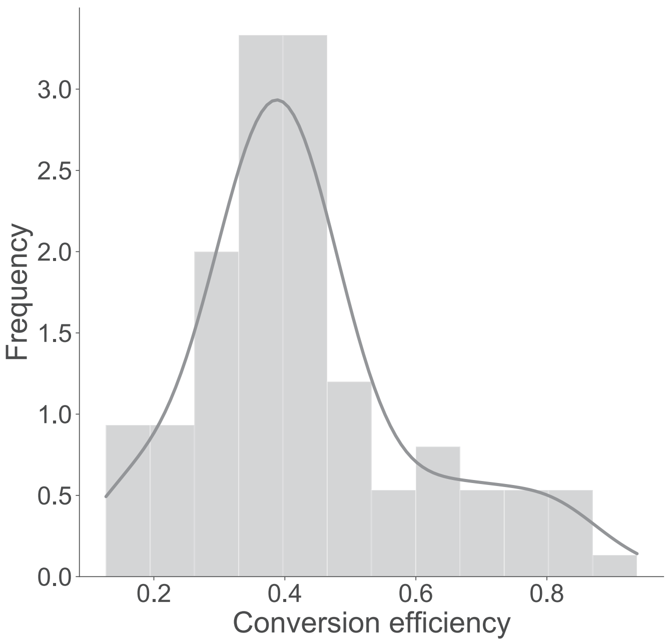

Supplementary Figure 1. Conversion efficiency for 111 clinical samples analyzed by the xF assay. The conversion efficiency was calculated as the median depth after removing duplicates divided by the minimum of the median depth before removing duplicates or the number of genome equivalents (based on 330 haploid genomes per ng input DNA). The median sample had a conversion efficiency of 40%.

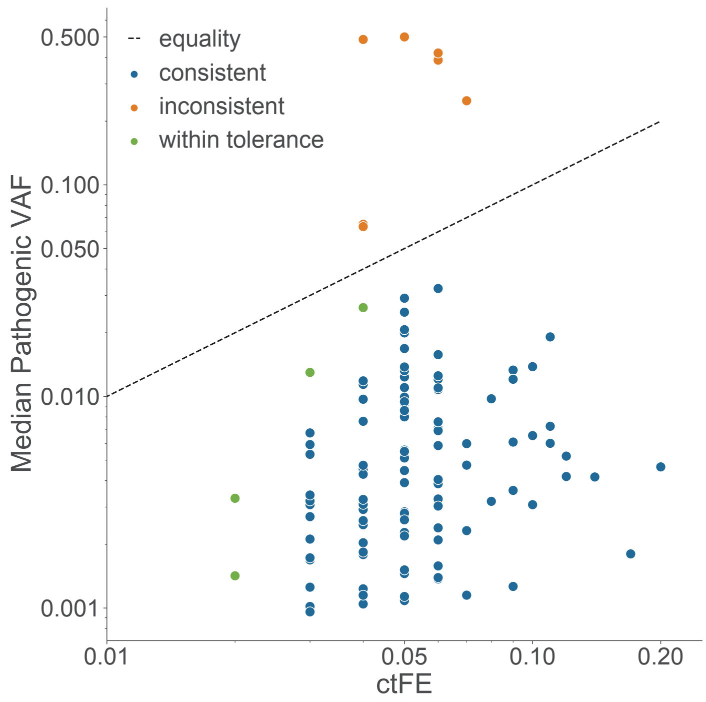

Supplementary Figure 2. Detected median VAFs are consistent with OTTER ctFEs in samples where ichorCNA estimates are zero. A variant can be detected at or below the tumor fraction of the sample, so if the median detected VAF is less than the OTTER ctFE, it is considered consistent.

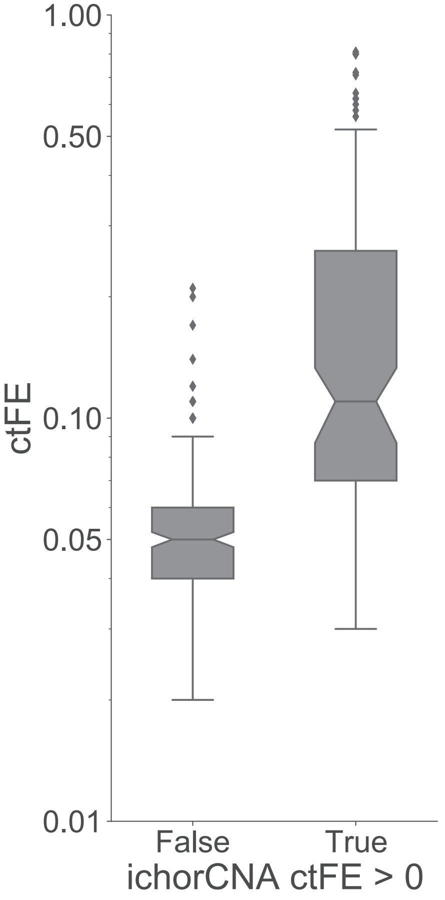

Supplementary Figure 3. Comparison of OTTER ctFEs when ichorCNA estimates from LPWGS are zero (n=210, median ctFE=0.05) or nonzero (n=165, media ctFE=0.11). The median OTTER ctFE is significantly higher (Mann-Whitney U test P=1.01e-27) when the tumor fraction is above the stated lower limit of detection 3%.

Supplementary Table 1. Analysis variants detected by the Tempus xF assay

Gene	SNV / Indel	Rearrangement	CNV
AKT1	•		
AKT2	•		
ALK	•	•	
APC	•		
AR	•		
ARAF	•		
ARID1A	•		
ATM	•		
ATR	•		
B2M	•		
BAP1	•		
BRAF	•	•	
BRCA1	•		
BRCA2	•		
BTK	•		
CCND1	•		
CCND2	•		
CCND3	•		
CCNE1	•		•
CD274	•		•
CDH1	•		
CDK4	•		
CDK6	•		
CDKN2A	•		
CTNNB1	•		
DDR2	•		
DPYD	•		
EGFR	•		•
ERBB2	•		•
ERRFI1	•		
ESR1	•		
EZH2	•		
FBXW7	•		
FGFR1	•		
FGFR2	•	•	
FGFR3	•	•	
FGFR4	•		
FLT3	•		
FOXL2	•		

GATA3	•		
GNA11	•		
GNAQ	•		
GNAS	•		
HNF1A	•		
HRAS	•		
IDH1	•		
IDH2	•		
JAK1	•		
JAK2	•		
JAK3	•		
KDR	•		
KEAP1	•		
KIT	•		
KMT2A	•		
KRAS	•		
MAP2K1	•		
MAP2K2	•		
MAPK1	•		
MET	•		•
MLH1	•		
MPL	•		
MSH2	•		
MSH3	•		
MSH6	•		
MTOR	•		
MYC	•		•
MYCN	•		
NF1	•		
NF2	•		
NFE2L2	•		
NOTCH1	•		
NPM1	•		
NRAS	•		
NTRK1	•	•	
PALB2	•		
PBRM1	•		
PDCD1LG2	•		
PDGFRA	•		
PDGFRB	•		
PIK3CA	•		
PIK3R1	•		
PMS2	•		

PTCH1	•	
PTEN	•	
PTPN11	•	
RAD51C	•	
RAF1	•	
RB1	•	
RET	•	•
RHEB	•	
RHOA	•	
RIT1	•	
RNF43	•	
ROS1	•	•
SDHA	•	
SMAD4	•	
SMO	•	
SPOP	•	
STK11	•	
TERT	•	
TP53	•	
TSC1	•	
TSC2	•	
UGT1A1	•	
VHL	•	

Supplementary Table 2. Overview of samples and experiments

Study	Number and Description of Samples
Validation: Limit of Detection	170 reference standard samples
Validation: Analytical Specificity	44 residual plasma samples
Validation: Inter-Instrument	10 blood specimens purchased from BioIVT (run in triplicate)
Validation: Inter-Assay	12 clinical samples
Validation: Intra-Assay	4 clinical samples (run in triplicate)
Validation: Analytical Accuracy	40 clinical samples run at 30ng and 29 clinical samples run at 10ng input DNA
Validation: Interfering Substances	12 clinical samples
Solid tumor assay comparison	55 matched blood and tumor samples
Microsatellite Instability Validation	65 blood samples
Low-pass whole-genome sequencing comparison	375 blood samples
Retrospective and clinical analyses	1,000 patient samples previously sequenced at Tempus Labs

Supplementary Table 3. Probabilistic noise filtering methodology improves variant calling

	Current Filtering Method	Dynamic Filtering Method	% Improvement Relative to Reference Samples
False Positive Variant Count	226	200	11.45%
True Positive Variant Count	164	162	-1.22%

Supplementary Table 4. Detailed overview of the concordance between the Tempus xF liquid biopsy and Tempus xT tissue assay

cancer_cohort	geneSymbol	pVar	cpra	allelicFraction	in_xT	in_xt_germ	xt_germ_is_filtered	in_xF	concordant
Non-Small Cell Lung Cancer	ATM	p.Asp1467Glu	11_108160493_C_A	0.023776224	TRUE	FALSE	FALSE	TRUE	TRUE
Non-Small Cell Lung Cancer	TP53	p.Thr253Pro	17_7577524_T_G	0.052954292	TRUE	FALSE	FALSE	TRUE	TRUE
Non-Small Cell Lung Cancer	KDR	p.Arg1232Gln	4_55948770_C_T	0.005263158	FALSE	TRUE	TRUE	TRUE	TRUE
Non-Small Cell Lung Cancer	KDR	p.Gln1225Leu	4_55948791_T_A	0.006650544	TRUE	FALSE	FALSE	TRUE	TRUE
Non-Small Cell Lung Cancer	MYC	p.Val117Glu	8_128750813_T_A	0.002822865	FALSE	FALSE	FALSE	TRUE	FALSE
Non-Small Cell Lung Cancer	CDKN2A	p.Glu61*	9_21971177_C_A	0.05141202	TRUE	FALSE	FALSE	TRUE	TRUE
Colorectal Cancer	TP53	p.Glu51*	17_7579536_C_A	0.183296784	TRUE	FALSE	FALSE	TRUE	TRUE
Colorectal Cancer	RNF43	p.Arg132*	17_56440943_G_A	0.179131886	TRUE	FALSE	FALSE	TRUE	TRUE
Colorectal Cancer	ALK	p.Ala298Ser	2_29917776_C_A	0.057582074	TRUE	TRUE	TRUE	TRUE	TRUE
Colorectal Cancer	BRAF	p.Val600Glu	7_140453136_A_T	0.24532921	TRUE	FALSE	FALSE	TRUE	TRUE
Colorectal Cancer	JAK2	p.Asp105Asn	9_5029869_G_A	0.242122952	TRUE	FALSE	FALSE	TRUE	TRUE
Non-Small Cell Lung Cancer	RB1	p.Arg857Pro	13_49050886_G_C	0.004750594	FALSE	TRUE	TRUE	TRUE	TRUE
Non-Small Cell Lung Cancer	TP53	p.His214Arg	17_7578208_T_C	0.002891102	FALSE	FALSE	FALSE	TRUE	FALSE
Non-Small Cell Lung Cancer	KDR	p.Glu284Gln	4_55979597_C_G	0.010298445	FALSE	FALSE	FALSE	TRUE	FALSE
Non-Small Cell Lung Cancer	SMO	p.Arg546Lys	7_128850374_G_A	0.004403439	FALSE	FALSE	FALSE	TRUE	FALSE
Colorectal Cancer	TP53	p.Cys176Phe	17_7578403_C_A	0.046700508	TRUE	FALSE	FALSE	TRUE	TRUE
Colorectal Cancer	APC	p.Arg805*	5_112173704_C_T	0.011996161	TRUE	FALSE	FALSE	TRUE	TRUE
Colorectal Cancer	APC	p.Arg1114*	5_112174631_C_T	0.028619529	TRUE	TRUE	TRUE	TRUE	TRUE
Colorectal Cancer	JAK2	p.Val366Met	9_5064922_G_A	0.478586279	FALSE	TRUE	FALSE	TRUE	TRUE
Colorectal Cancer	ATM	p.Leu15Gln	11_108098395_T_A	0.029057931	TRUE	FALSE	FALSE	TRUE	TRUE
Colorectal Cancer	ATM	p.Leu715Val	11_108126960_C_G	0.00499002	FALSE	TRUE	TRUE	TRUE	TRUE
Colorectal Cancer	KRAS	p.Ala146Thr	12_25378562_C_T	0.038065844	TRUE	FALSE	FALSE	TRUE	TRUE
Colorectal Cancer	NF1	p.Phe738fs	17_29553663_T_TAA	0.011989101	FALSE	TRUE	TRUE	TRUE	TRUE
Colorectal Cancer	IDH1	p.Arg132Cys	2_209113113_G_A	0.031975151	TRUE	FALSE	FALSE	TRUE	TRUE
Colorectal Cancer	PIK3CA	p.Glu542Lys	3_178936082_G_A	0.024231371	TRUE	FALSE	FALSE	TRUE	TRUE
Colorectal Cancer	PDGFRA	p.Glu156Lys	4_55129932_G_A	0.482218684	FALSE	TRUE	FALSE	TRUE	TRUE
Colorectal Cancer	APC	p.Ser1465fs	5_112175675_AAG_A	0.059882439	TRUE	TRUE	TRUE	TRUE	TRUE
Breast Cancer	ARID1A	p.Gly779*	1_27088726_G_T	0.242402374	TRUE	FALSE	FALSE	TRUE	TRUE
Breast Cancer	KMT2A	p.Ser215Pro	11_118342517_T_C	0.445657631	FALSE	TRUE	FALSE	TRUE	TRUE
Breast Cancer	RB1	p.Trp75fs	13_48881499_C_CT	0.007437186	FALSE	FALSE	FALSE	TRUE	FALSE
Breast Cancer	RB1	p.Ser350Ile	13_48941739_G_T	0.022074136	FALSE	FALSE	FALSE	TRUE	FALSE
Breast Cancer	RB1	p.Arg455*	13_48953760_C_T	0.016976127	TRUE	FALSE	FALSE	TRUE	TRUE

Breast Cancer	RB1	p.Met484fs	13_48954326_CAT_C	0.026795284	FALSE	FALSE	FALSE	TRUE	FALSE
Breast Cancer	RB1	p.Gln637*	13_49030434_C_T	0.028092367	FALSE	FALSE	FALSE	TRUE	FALSE
Breast Cancer	RB1	p.Leu665Pro	13_49033857_T_C	0.010609358	FALSE	FALSE	FALSE	TRUE	FALSE
Breast Cancer	RB1	p.Gln736*	13_49037966_C_T	0.124695699	FALSE	FALSE	FALSE	TRUE	FALSE
Breast Cancer	RB1	p.Lys740*	13_49039140_A_T	0.011514615	FALSE	FALSE	FALSE	TRUE	FALSE
Breast Cancer	RB1	p.Gln770*	13_49039230_C_T	0.006858711	FALSE	FALSE	FALSE	TRUE	FALSE
Breast Cancer	TP53	p.Arg209fs	17_7578221_TTC_T	0.260776782	TRUE	TRUE	TRUE	TRUE	TRUE
Breast Cancer	NF1	p.Glu1088Gln	17_29559155_G_C	0.153587184	TRUE	FALSE	FALSE	TRUE	TRUE
Breast Cancer	NF2	p.Asp508Asn	22_30074260_G_A	0.597653293	FALSE	TRUE	FALSE	TRUE	TRUE
Breast Cancer	BAP1	p.Arg114His	3_52442008_C_T	0.25955774	FALSE	TRUE	FALSE	TRUE	TRUE
Colorectal Cancer	ARID1A	p.Pro453Ser	1_27057649_C_T	0.460788297	FALSE	TRUE	FALSE	TRUE	TRUE
Colorectal Cancer	BRCA2	p.Gly1376Ala	13_32912619_G_C	0.003231018	FALSE	FALSE	FALSE	TRUE	FALSE
Colorectal Cancer	TP53	p.Tyr220Cys	17_7578190_T_C	0.003069577	FALSE	FALSE	FALSE	TRUE	FALSE
Colorectal Cancer	NF1	p.Asn916Ser	17_29556380_A_G	0.003615002	FALSE	FALSE	FALSE	TRUE	FALSE
Colorectal Cancer	FGFR3	0	4_1809619_G_T	0.004125129	FALSE	FALSE	FALSE	TRUE	FALSE
Colorectal Cancer	SMO	p.Asn309Ser	7_128845996_A_G	0.02092737	FALSE	FALSE	FALSE	TRUE	FALSE
Colorectal Cancer	CDKN2A	p.Ser12Leu	9_21974792_G_A	0.003692762	FALSE	FALSE	FALSE	TRUE	FALSE
Colorectal Cancer	DDR2	p.Glu658Asp	1_162745559_G_T	0.010059097	FALSE	FALSE	FALSE	TRUE	FALSE
Colorectal Cancer	KRAS	p.Gln61His	12_25380275_T_G	0.004399413	FALSE	FALSE	FALSE	TRUE	FALSE
Colorectal Cancer	KRAS	p.Gly12Cys	12_25398285_C_A	0.013344739	FALSE	FALSE	FALSE	TRUE	FALSE
Colorectal Cancer	TP53	p.Gln104*	17_7579377_G_A	0.593439097	TRUE	FALSE	FALSE	TRUE	TRUE
Colorectal Cancer	SMAD4	p.Pro356Leu	18_48591904_C_T	0.46580114	TRUE	FALSE	FALSE	TRUE	TRUE
Colorectal Cancer	MSH2	p.Met453Ile	2_47672769_G_A	0.005264052	FALSE	FALSE	FALSE	TRUE	FALSE
Colorectal Cancer	BAP1	p.Trp196Ser	3_52440917_C_G	0.006391235	FALSE	TRUE	TRUE	TRUE	TRUE
Colorectal Cancer	PDGFRA	p.Asn468Thr	4_55139742_A_C	0.002693401	FALSE	FALSE	FALSE	TRUE	FALSE
Colorectal Cancer	APC	p.Thr273fs	5_112137061_CA_C	0.456933759	TRUE	FALSE	FALSE	TRUE	TRUE
Colorectal Cancer	KMT2A	p.Asn3797Tyr	11_118390739_A_T	0.002527486	FALSE	FALSE	FALSE	TRUE	FALSE
Colorectal Cancer	KRAS	p.Gly13Asp	12_25398281_C_T	0.295867251	TRUE	FALSE	FALSE	TRUE	TRUE
Colorectal Cancer	TP53	p.Arg283Pro	17_7577089_GC_AG	0.345970342	TRUE	FALSE	FALSE	TRUE	TRUE
Colorectal Cancer	APC	p.Arg1450*	5_112175639_C_T	0.181946403	TRUE	FALSE	FALSE	TRUE	TRUE
Colorectal Cancer	MYC	p.Ala200Thr	8_128751061_G_A	0.211214118	TRUE	FALSE	FALSE	TRUE	TRUE
Colorectal Cancer	CDKN2A	p.Gly67Ser	9_21971159_C_T	0.063066721	FALSE	FALSE	FALSE	TRUE	FALSE
Colorectal Cancer	RB1	p.Tyr813*	13_49039454_T_G	0.221986908	FALSE	TRUE	TRUE	TRUE	TRUE
Colorectal Cancer	ATM	p.Ile1740Phe	11_108172415_A_T	0.005278116	FALSE	TRUE	TRUE	TRUE	TRUE

Colorectal Cancer	MSH3	p.Ala68_Pro69ins ProProAlaProPro AlaProProAla	5_79950727_G_GCAG GCCCCCAGCGCCCC CAGCGCCCC	0.121540313	FALSE	FALSE	FALSE	TRUE	FALSE
Colorectal Cancer	EGFR	p.Pro385Ser	7_55224471_C_T	0.002707407	FALSE	FALSE	FALSE	TRUE	FALSE
Colorectal Cancer	FGFR1	p.Val38Met	8_38287446_C_T	0.463992909	FALSE	TRUE	FALSE	TRUE	TRUE
Colorectal Cancer	MYC	p.Ser151Asn	8_128750915_G_A	0.494300046	FALSE	TRUE	FALSE	TRUE	TRUE
Colorectal Cancer	MTOR	p.Gly1822Arg	1_11190735_C_T	0.007600793	TRUE	TRUE	TRUE	TRUE	TRUE
Colorectal Cancer	ARID1A	p.Arg1721*	1_27105550_C_T	0.009617818	TRUE	FALSE	FALSE	TRUE	TRUE
Colorectal Cancer	NTRK1	p.Leu700Met	1_156849842_C_A	0.004606847	TRUE	FALSE	FALSE	TRUE	TRUE
Colorectal Cancer	ATM	p.Leu443Ile	11_108121519_C_A	0.011043742	TRUE	FALSE	FALSE	TRUE	TRUE
Colorectal Cancer	ATM	p.Ala2883Val	11_108218069_C_T	0.004560603	TRUE	TRUE	TRUE	TRUE	TRUE
Colorectal Cancer	CCND2	p.Asn150Ser	12_4387963_A_G	0.016679749	FALSE	FALSE	FALSE	TRUE	FALSE
Colorectal Cancer	KRAS	p.Gly12Asp	12_25398284_C_T	0.022811598	TRUE	FALSE	FALSE	TRUE	TRUE
Colorectal Cancer	B2M	p.Cys45*	15_45007688_C_A	0.011234226	TRUE	TRUE	TRUE	TRUE	TRUE
Colorectal Cancer	PALB2	p.Pro473Ser	16_23646450_G_A	0.003775261	FALSE	FALSE	FALSE	TRUE	FALSE
Colorectal Cancer	TP53	p.Arg175His	17_7578406_C_T	0.014778922	TRUE	FALSE	FALSE	TRUE	TRUE
Colorectal Cancer	STK11	p.Ala397Val	19_1226534_C_T	0.010713237	TRUE	FALSE	FALSE	TRUE	TRUE
Colorectal Cancer	MSH2	p.Arg534Cys	2_47693886_C_T	0.012866137	TRUE	FALSE	FALSE	TRUE	TRUE
Colorectal Cancer	MSH6	p.Leu979Pro	2_48028058_T_C	0.493133295	FALSE	TRUE	FALSE	TRUE	TRUE
Colorectal Cancer	PIK3CA	p.His1047Arg	3_178952085_A_G	0.009610134	TRUE	FALSE	FALSE	TRUE	TRUE
Colorectal Cancer	CDKN2A	p.Arg58*	9_21971186_G_A	0.005773132	TRUE	FALSE	FALSE	TRUE	TRUE
Colorectal Cancer	PTCH1	p.Arg1284Gln	9_98209489_C_T	0.006836308	TRUE	FALSE	FALSE	TRUE	TRUE
Colorectal Cancer	PTCH1	p.Val482Met	9_98238402_C_T	0.011024469	TRUE	FALSE	FALSE	TRUE	TRUE
Colorectal Cancer	NOTCH1	p.Arg1234Gln	9_139401368_C_T	0.011294649	TRUE	FALSE	FALSE	TRUE	TRUE
Colorectal Cancer	RET	p.Arg693Cys	10_43610125_C_T	0.027872635	TRUE	FALSE	FALSE	TRUE	TRUE
Colorectal Cancer	RET	p.Val804Met	10_43614996_G_A	0.464314036	TRUE	TRUE	FALSE	TRUE	TRUE
Colorectal Cancer	KRAS	p.Gly12Cys	12_25398285_C_A	0.024973188	TRUE	FALSE	FALSE	TRUE	TRUE
Colorectal Cancer	TP53	p.Arg280Thr	17_7577099_C_G	0.029379892	TRUE	FALSE	FALSE	TRUE	TRUE
Breast Cancer	CCND1	p.Ser41Leu	11_69456203_C_T	0.003028009	FALSE	FALSE	FALSE	TRUE	FALSE
Breast Cancer	ATM	p.Arg2191Gly	11_108192146_A_G	0.490272374	FALSE	TRUE	FALSE	TRUE	TRUE
Breast Cancer	TSC2	p.Glu158Asp	16_2104434_A_C	0.003270111	FALSE	FALSE	FALSE	TRUE	FALSE
Breast Cancer	RAD51C	p.Pro21Ser	17_56770065_C_T	0.004112808	TRUE	FALSE	FALSE	TRUE	TRUE
Breast Cancer	MLH1	p.Arg9Trp	3_37035063_C_T	0.003579952	FALSE	FALSE	FALSE	TRUE	FALSE
Breast Cancer	PIK3CA	p.His1047Arg	3_178952085_A_G	0.014295758	TRUE	FALSE	FALSE	TRUE	TRUE
Breast Cancer	LETM1	0	4_1808473_G_A	0.008587377	FALSE	FALSE	FALSE	TRUE	FALSE
Breast Cancer	MET	p.Cys1210Ser	7_116422148_G_C	0.002559415	FALSE	FALSE	FALSE	TRUE	FALSE
Non-Small Cell Lung Cancer	ROS1	p.Ala347Val	6_117715449_G_A	0.00817757	FALSE	TRUE	TRUE	TRUE	TRUE
Non-Small Cell Lung Cancer	MYC	p.Asn24Ser	8_128750534_A_G	0.522119592	FALSE	TRUE	FALSE	TRUE	TRUE

Non-Small Cell Lung Cancer	NOTCH1	p.Pro407Leu	9_139412624_G_A	0.495975855	FALSE	TRUE	FALSE	TRUE	TRUE
Non-Small Cell Lung Cancer	AR	p.Thr851Ser	X_66942770_A_T	0.003125	FALSE	FALSE	FALSE	TRUE	FALSE
Breast Cancer	FGFR1	p.Met456Ile	8_38275808_C_A	0.501705902	FALSE	TRUE	FALSE	TRUE	TRUE
Breast Cancer	GATA3	p.Pro409fs	10_8115874_C_CG	0.029266573	TRUE	FALSE	FALSE	TRUE	TRUE
Breast Cancer	TP53	p.Arg249Ser	17_7577534_C_G	0.030864198	TRUE	FALSE	FALSE	TRUE	TRUE
Breast Cancer	FGFR1	p.Lys656Glu	8_38272308_T_C	0.020485584	FALSE	FALSE	FALSE	TRUE	FALSE
Non-Small Cell Lung Cancer	RET	p.Asp577Tyr	10_43608381_G_T	0.005842525	FALSE	FALSE	FALSE	TRUE	FALSE
Non-Small Cell Lung Cancer	RET	p.Glu616Gln	10_43609090_G_C	0.010081156	FALSE	FALSE	FALSE	TRUE	FALSE
Non-Small Cell Lung Cancer	RET	p.Val685Phe	10_43610101_G_T	0.005725091	FALSE	FALSE	FALSE	TRUE	FALSE
Non-Small Cell Lung Cancer	TP53	p.Cys275Phe	17_7577114_C_A	0.60507771	TRUE	TRUE	TRUE	TRUE	TRUE
Non-Small Cell Lung Cancer	ERBB2	p.Asp1016Tyr	17_37883143_G_T	0.002993892	FALSE	FALSE	FALSE	TRUE	FALSE
Non-Small Cell Lung Cancer	PMS2	p.Glu44Gln	7_6045556_C_G	0.451980372	FALSE	TRUE	FALSE	TRUE	TRUE
Non-Small Cell Lung Cancer	EGFR	p.Leu718Val	7_55241704_C_G	0.005783239	FALSE	FALSE	FALSE	TRUE	FALSE
Non-Small Cell Lung Cancer	EGFR	p.Leu858Arg	7_55259515_T_G	0.900951745	TRUE	TRUE	TRUE	TRUE	TRUE
Non-Small Cell Lung Cancer	MET	p.Ala271Thr	7_116339949_G_A	0.364584726	FALSE	TRUE	FALSE	TRUE	TRUE
Non-Small Cell Lung Cancer	GATA3	p.Arg276Gln	10_8106004_G_A	0.004295391	TRUE	FALSE	FALSE	TRUE	TRUE
Non-Small Cell Lung Cancer	KRAS	p.Gly12Cys	12_25398285_C_A	0.005798728	TRUE	FALSE	FALSE	TRUE	TRUE
Non-Small Cell Lung Cancer	ALK	p.Gly306Trp	2_29917752_C_A	0.00392773	TRUE	FALSE	FALSE	TRUE	TRUE
Non-Small Cell Lung Cancer	MSH6	p.Gly31Ser	2_48010463_G_A	0.540669856	FALSE	TRUE	FALSE	TRUE	TRUE
Non-Small Cell Lung Cancer	KDR	p.Ile624Leu	4_55970927_T_G	0.002680638	TRUE	FALSE	FALSE	TRUE	TRUE
Breast Cancer	RB1	p.Leu317Pro	13_48941640_T_C	0.004245465	FALSE	FALSE	FALSE	TRUE	FALSE
Breast Cancer	TP53	p.Gly245Asp	17_7577547_C_T	0.447754137	TRUE	TRUE	TRUE	TRUE	TRUE
Breast Cancer	BAP1	p.Val439Leu	3_52437846_C_A	0.492199554	TRUE	FALSE	FALSE	TRUE	TRUE
Breast Cancer	DDR2	p.Asp472Asn	1_162740212_G_A	0.003533916	FALSE	FALSE	FALSE	TRUE	FALSE
Breast Cancer	ATM	p.Arg925fs	11_108139268_CG_C	0.007215207	FALSE	TRUE	TRUE	TRUE	TRUE
Breast Cancer	TP53	p.Glu56*	17_7579521_C_A	0.07023185	TRUE	FALSE	FALSE	TRUE	TRUE
Breast Cancer	KIT	p.Asn293Ser	4_55570011_A_G	0.478423625	FALSE	TRUE	FALSE	TRUE	TRUE
Colorectal Cancer	KRAS	p.Gly12Val	12_25398284_C_A	0.458608059	TRUE	FALSE	FALSE	TRUE	TRUE
Colorectal Cancer	TP53	p.Pro177_Cys18[illegible] del	17_7578383_AGCAGC[illegible] CTCATGGTGGGG_A	0.580955072	TRUE	FALSE	FALSE	TRUE	TRUE
Colorectal Cancer	SMAD4	p.Gly419Trp	18_48593504_G_T	0.424114671	TRUE	FALSE	FALSE	TRUE	TRUE
Colorectal Cancer	TERT	p.Gly42Arg	5_1294981_C_T	0.012658228	FALSE	FALSE	FALSE	TRUE	FALSE
Colorectal Cancer	APC	p.Tyr1179fs	5_112174826_TA_T	0.062908598	FALSE	FALSE	FALSE	TRUE	FALSE
Colorectal Cancer	APC	p.Arg1450*	5_112175639_C_T	0.585497252	TRUE	FALSE	FALSE	TRUE	TRUE
Non-Small Cell Lung Cancer	DDR2	p.Ile474Val	1_162740218_A_G	0.021909633	FALSE	FALSE	FALSE	TRUE	FALSE
Non-Small Cell Lung Cancer	RET	p.Arg721Trp	10_43612056_C_T	0.002992958	FALSE	FALSE	FALSE	TRUE	FALSE
Non-Small Cell Lung Cancer	ATM	p.Arg1875*	11_108175528_C_T	0.002612571	FALSE	FALSE	FALSE	TRUE	FALSE

Non-Small Cell Lung Cancer	KMT2A	p.Arg2659Gln	11_118374583_G_A	0.002892718	FALSE	FALSE	FALSE	TRUE	FALSE
Non-Small Cell Lung Cancer	RB1	p.Met484fs	13_48954326_CAT_C	0.025841817	FALSE	FALSE	FALSE	TRUE	FALSE
Non-Small Cell Lung Cancer	TP53	p.Arg273Cys	17_7577121_G_A	0.36799869	TRUE	FALSE	FALSE	TRUE	TRUE
Non-Small Cell Lung Cancer	ERBB2	p.Arg103Gln	17_37864656_G_A	0.005242464	FALSE	FALSE	FALSE	TRUE	FALSE
Non-Small Cell Lung Cancer	RNF43	p.Arg113*	17_56448310_G_A	0.0025357	FALSE	FALSE	FALSE	TRUE	FALSE
Non-Small Cell Lung Cancer	MLH1	p.Ser368Leu	3_37067192_C_T	0.005414185	FALSE	FALSE	FALSE	TRUE	FALSE
Non-Small Cell Lung Cancer	CDKN2A	p.Ser12Leu	9_21974792_G_A	0.003334344	FALSE	FALSE	FALSE	TRUE	FALSE
Non-Small Cell Lung Cancer	NOTCH1	p.Glu1636Lys	9_139399237_C_T	0.004282655	FALSE	FALSE	FALSE	TRUE	FALSE
Colorectal Cancer	KRAS	p.Gly12Asp	12_25398284_C_T	0.053145336	TRUE	FALSE	FALSE	TRUE	TRUE
Colorectal Cancer	TP53	p.His193Leu	17_7578271_T_A	0.024947146	TRUE	FALSE	FALSE	TRUE	TRUE
Colorectal Cancer	FGFR3	0	4_1809908_GGA_G	0.487945493	FALSE	TRUE	FALSE	TRUE	TRUE
Colorectal Cancer	APC	p.Ser1465fs	5_112175675_A_AAG	0.040908359	TRUE	FALSE	FALSE	TRUE	TRUE
Colorectal Cancer	NOTCH1	p.Pro2514fs	9_139390648_CAG_C	0.01029552	FALSE	FALSE	FALSE	TRUE	FALSE
Colorectal Cancer	AR	p.Arg616His	X_66905930_G_A	0.042466313	TRUE	FALSE	FALSE	TRUE	TRUE
Breast Cancer	ERRFI1	p.Glu408Lys	1_8073437_C_T	0.038033829	FALSE	FALSE	FALSE	TRUE	FALSE
Breast Cancer	ARID1A	p.Gln633*	1_27059260_C_T	0.006066223	FALSE	FALSE	FALSE	TRUE	FALSE
Breast Cancer	GATA3	p.Ser137Leu	10_8100436_C_T	0.002504871	FALSE	FALSE	FALSE	TRUE	FALSE
Breast Cancer	PTEN	p.Asn12fs	10_89624261_ACAAAAGG_A	0.00509725	FALSE	FALSE	FALSE	TRUE	FALSE
Breast Cancer	HRAS	p.Asp119His	11_533548_C_G	0.016099635	FALSE	FALSE	FALSE	TRUE	FALSE
Breast Cancer	HRAS	p.Gly13Val	11_534285_C_A	0.003922138	TRUE	FALSE	FALSE	TRUE	TRUE
Breast Cancer	KRAS	p.Gly12Asp	12_25398284_C_T	0.037796374	FALSE	FALSE	FALSE	TRUE	FALSE
Breast Cancer	CDK4	p.Ser189Phe	12_58144505_G_A	0.003557532	TRUE	FALSE	FALSE	TRUE	TRUE
Breast Cancer	FLT3	p.Arg707Lys	13_28601312_C_T	0.022736233	FALSE	FALSE	FALSE	TRUE	FALSE
Breast Cancer	RB1	p.Tyr498fs	13_48954371_TA_T	0.237942979	TRUE	FALSE	FALSE	TRUE	TRUE
Breast Cancer	B2M	p.Met1?	15_45003747_G_A	0.041005291	TRUE	FALSE	FALSE	TRUE	TRUE
Breast Cancer	TSC2	p.Ser739Tyr	16_2122360_C_A	0.004127411	FALSE	FALSE	FALSE	TRUE	FALSE
Breast Cancer	TSC2	p.Arg1138*	16_2130180_C_T	0.0067963	FALSE	FALSE	FALSE	TRUE	FALSE
Breast Cancer	PALB2	p.Lys695Asn	16_23641390_C_G	0.002953392	FALSE	FALSE	FALSE	TRUE	FALSE
Breast Cancer	PALB2	p.Glu658Lys	16_23641503_C_T	0.050882752	FALSE	FALSE	FALSE	TRUE	FALSE
Breast Cancer	CDH1	p.Ser36fs	16_68772255_AG_A	0.096506809	TRUE	FALSE	FALSE	TRUE	TRUE
Breast Cancer	TP53	p.Arg280Thr	17_7577099_C_G	0.008467121	FALSE	FALSE	FALSE	TRUE	FALSE
Breast Cancer	TP53	p.Ser241Cys	17_7577559_G_C	0.045225427	FALSE	FALSE	FALSE	TRUE	FALSE
Breast Cancer	TP53	p.Glu224Lys	17_7578179_C_T	0.024728997	TRUE	FALSE	FALSE	TRUE	TRUE
Breast Cancer	NF1	p.Ser666Cys	17_29552264_C_G	0.018500721	FALSE	FALSE	FALSE	TRUE	FALSE
Breast Cancer	NF1	p.Lys2535Asn	17_29679422_G_C	0.028021248	TRUE	FALSE	FALSE	TRUE	TRUE
Breast Cancer	BRCA1	p.Ser1007*	17_41244528_G_T	0.005556173	TRUE	TRUE	TRUE	TRUE	TRUE
Breast Cancer	BRCA1	p.Gln910Glu	17_41244820_G_C	0.004162607	TRUE	FALSE	FALSE	TRUE	TRUE

Breast Cancer	BRCA1	p.Ser561Cys	17_41245866_G_C	0.004253779	TRUE	FALSE	FALSE	TRUE	TRUE
Breast Cancer	RNF43	p.Asp506His	17_56435621_C_G	0.1202954	FALSE	FALSE	FALSE	TRUE	FALSE
Breast Cancer	SMAD4	p.Gly352Glu	18_48591892_G_A	0.006232175	FALSE	FALSE	FALSE	TRUE	FALSE
Breast Cancer	SMAD4	p.Pro356His	18_48591904_C_A	0.023255814	FALSE	FALSE	FALSE	TRUE	FALSE
Breast Cancer	SMAD4	p.Arg361His	18_48591919_G_A	0.033848176	FALSE	FALSE	FALSE	TRUE	FALSE
Breast Cancer	SMAD4	p.Cys363Phe	18_48591925_G_T	0.004638834	FALSE	FALSE	FALSE	TRUE	FALSE
Breast Cancer	SMAD4	p.Asp493Asn	18_48604655_G_A	0.009721757	FALSE	FALSE	FALSE	TRUE	FALSE
Breast Cancer	SMAD4	p.Glu526Lys	18_48604754_G_A	0.054857756	FALSE	FALSE	FALSE	TRUE	FALSE
Breast Cancer	MSH6	p.Pro408Ser	2_48026344_C_T	0.119221652	FALSE	FALSE	FALSE	TRUE	FALSE
Breast Cancer	MSH6	p.Pro898Ser	2_48027814_C_T	0.003577818	TRUE	FALSE	FALSE	TRUE	TRUE
Breast Cancer	MSH6	p.Ser1246Leu	2_48033433_C_T	0.018682748	FALSE	FALSE	FALSE	TRUE	FALSE
Breast Cancer	GNAS	p.Arg201Cys	20_57484420_C_T	0.058795638	FALSE	TRUE	TRUE	TRUE	TRUE
Breast Cancer	PBRM1	p.Arg761Lys	3_52643614_C_T	0.00331565	FALSE	FALSE	FALSE	TRUE	FALSE
Breast Cancer	ATR	p.Phe1033Val	3_142268395_A_C	0.003534818	FALSE	FALSE	FALSE	TRUE	FALSE
Breast Cancer	ATR	p.Gln713Glu	3_142274923_G_C	0.043441407	FALSE	FALSE	FALSE	TRUE	FALSE
Breast Cancer	PIK3CA	p.Glu39Lys	3_178916728_G_A	0.120815304	FALSE	FALSE	FALSE	TRUE	FALSE
Breast Cancer	PIK3CA	p.Glu418Lys	3_178927974_G_A	0.008615385	FALSE	FALSE	FALSE	TRUE	FALSE
Breast Cancer	PIK3CA	p.His1047Arg	3_178952085_A_G	0.155564341	TRUE	FALSE	FALSE	TRUE	TRUE
Breast Cancer	FGFR3	p.Ile285Met	4_1803677_C_G	0.04794169	FALSE	FALSE	FALSE	TRUE	FALSE
Breast Cancer	KIT	p.Arg796Lys	4_55599261_G_A	0.136429819	FALSE	FALSE	FALSE	TRUE	FALSE
Breast Cancer	KIT	p.Glu898Lys	4_55602982_G_A	0.00391252	TRUE	FALSE	FALSE	TRUE	TRUE
Breast Cancer	FBXW7	p.Gln3Glu	4_153332949_G_C	0.026638559	TRUE	FALSE	FALSE	TRUE	TRUE
Breast Cancer	TERT	0	5_1295105_C_G	0.008944544	FALSE	FALSE	FALSE	TRUE	FALSE
Breast Cancer	APC	p.Glu287Lys	5_112151216_G_A	0.119464945	FALSE	FALSE	FALSE	TRUE	FALSE
Breast Cancer	APC	p.Asp808His	5_112173713_G_C	0.007465544	FALSE	FALSE	FALSE	TRUE	FALSE
Breast Cancer	APC	p.Gln1406His	5_112175509_G_C	0.025126098	TRUE	FALSE	FALSE	TRUE	TRUE
Breast Cancer	APC	p.Glu1645Lys	5_112176224_G_A	0.004370152	TRUE	FALSE	FALSE	TRUE	TRUE
Breast Cancer	APC	p.Glu1998Lys	5_112177283_G_A	0.046237364	FALSE	FALSE	FALSE	TRUE	FALSE
Breast Cancer	APC	p.Asp2490His	5_112178759_G_C	0.023027523	TRUE	FALSE	FALSE	TRUE	TRUE
Breast Cancer	FGFR4	p.Val550Met	5_176522551_G_A	0.055242545	FALSE	FALSE	FALSE	TRUE	FALSE
Breast Cancer	CCND3	p.Ala230Val	6_41904319_G_A	0.00286533	FALSE	FALSE	FALSE	TRUE	FALSE
Breast Cancer	CCND3	p.Ser221Phe	6_41904346_G_A	0.031764256	TRUE	FALSE	FALSE	TRUE	TRUE
Breast Cancer	ROS1	p.Gln2160*	6_117630048_G_A	0.131966117	FALSE	FALSE	FALSE	TRUE	FALSE
Breast Cancer	EGFR	p.Gly459Ala	7_55227909_G_C	0.003106441	FALSE	FALSE	FALSE	TRUE	FALSE
Breast Cancer	EGFR	p.Glu709Lys	7_55241677_G_A	0.007283633	FALSE	FALSE	FALSE	TRUE	FALSE
Breast Cancer	MET	p.Glu1172Gln	7_116419003_G_C	0.01716052	TRUE	FALSE	FALSE	TRUE	TRUE
Breast Cancer	MET	p.Pro1285Leu	7_116435764_C_T	0.01324261	FALSE	FALSE	FALSE	TRUE	FALSE
Breast Cancer	MYC	p.Glu424Lys	8_128753109_G_A	0.017984916	FALSE	FALSE	FALSE	TRUE	FALSE

Breast Cancer	GNAQ	p.Glu212Gln	9_80409480_C_G	0.005090909	TRUE	FALSE	FALSE	TRUE	TRUE
Breast Cancer	NOTCH1	p.Lys1774Arg	9_139396787_T_C	0.011245229	FALSE	FALSE	FALSE	TRUE	FALSE
Breast Cancer	NOTCH1	p.Ser402Leu	9_139412639_G_A	0.020108184	TRUE	FALSE	FALSE	TRUE	TRUE
Non-Small Cell Lung Cancer	KRAS	p.Gly12Asp	12_25398284_C_T	0.07940684	TRUE	FALSE	FALSE	TRUE	TRUE
Non-Small Cell Lung Cancer	TP53	p.Val157Phe	17_7578461_C_A	0.079240037	TRUE	FALSE	FALSE	TRUE	TRUE
Non-Small Cell Lung Cancer	ERBB2	p.Lys228Asn	17_37866379_G_T	0.032129404	TRUE	TRUE	TRUE	TRUE	TRUE
Non-Small Cell Lung Cancer	STK11	p.Gln37*	19_1207021_C_T	0.067213755	TRUE	FALSE	FALSE	TRUE	TRUE
Colorectal Cancer	MTOR	p.Ala519Thr	1_11300591_C_T	0.495206612	FALSE	TRUE	FALSE	TRUE	TRUE
Colorectal Cancer	PDGFRA	p.Leu47Arg	4_55127352_T_G	0.002839565	FALSE	FALSE	FALSE	TRUE	FALSE
Colorectal Cancer	MSH3	p.Ala68_Pro69ins ProProAlaProPro AlaProProAla	5_79950727_G_GCAG(GCCCCCAGCGCCCC CAGCGCCCC	0.095890411	FALSE	FALSE	FALSE	TRUE	FALSE
Colorectal Cancer	APC	p.Arg876*	5_112173917_C_T	0.002678401	TRUE	FALSE	FALSE	TRUE	TRUE
Colorectal Cancer	ESR1	p.ProIle325ProVa l	6_152265522_GA_CG	0.508278521	FALSE	FALSE	FALSE	TRUE	FALSE
Colorectal Cancer	EGFR	p.Leu858Arg	7_55259515_T_G	0.004866695	TRUE	TRUE	TRUE	TRUE	TRUE
Breast Cancer	RET	p.Val455Ile	10_43606754_G_A	0.511836359	FALSE	TRUE	FALSE	TRUE	TRUE
Breast Cancer	TP53	p.Phe113Val	17_7579350_A_C	0.121663313	TRUE	FALSE	FALSE	TRUE	TRUE
Breast Cancer	PIK3CA	p.HisHis1047Arg Arg	3_178952085_ATCA_G CG	0.171113831	TRUE	FALSE	FALSE	TRUE	TRUE
Breast Cancer	ERRFI1	p.Ser330Leu	1_8073670_G_A	0.288103906	TRUE	TRUE	TRUE	TRUE	TRUE
Breast Cancer	BRCA2	p.Gly1376Ala	13_32912619_G_C	0.005524862	FALSE	FALSE	FALSE	TRUE	FALSE
Breast Cancer	RB1	p.Ser178fs	13_48921988_AC_A	0.006911604	FALSE	FALSE	FALSE	TRUE	FALSE
Breast Cancer	RB1	p.Ser634*	13_49030426_C_G	0.139523254	FALSE	FALSE	FALSE	TRUE	FALSE
Breast Cancer	TP53	p.Gln136Glu	17_7578524_G_C	0.374180251	TRUE	FALSE	FALSE	TRUE	TRUE
Breast Cancer	ERBB2	p.Gly776Val	17_37880998_G_T	0.016524083	FALSE	FALSE	FALSE	TRUE	FALSE
Breast Cancer	PBRM1	p.Glu449Gln	3_52663008_C_G	0.003368338	FALSE	FALSE	FALSE	TRUE	FALSE
Breast Cancer	PIK3CA	p.Asn1068fs	3_178952146_G_GA	0.740670462	TRUE	FALSE	FALSE	TRUE	TRUE
Breast Cancer	ESR1	p.Glu380Gln	6_152332832_G_C	0.006756757	FALSE	FALSE	FALSE	TRUE	FALSE
Colorectal Cancer	TP53	p.Arg249Ser	17_7577534_C_G	0.003109815	TRUE	FALSE	FALSE	TRUE	TRUE
Colorectal Cancer	ALK	p.Arg1275*	2_29432665_G_A	0.008200754	FALSE	FALSE	FALSE	TRUE	FALSE
Colorectal Cancer	FGFR3	0	4_1809253_G_A	0.470244238	FALSE	TRUE	FALSE	TRUE	TRUE
Colorectal Cancer	KRAS	p.Gly12Asp	12_25398284_C_T	0.077151335	TRUE	FALSE	FALSE	TRUE	TRUE
Colorectal Cancer	SMAD4	p.Pro130Leu	18_48575195_C_T	0.037112637	TRUE	FALSE	FALSE	TRUE	TRUE
Colorectal Cancer	SMAD4	p.Arg361His	18_48591919_G_A	0.038696109	TRUE	FALSE	FALSE	TRUE	TRUE
Colorectal Cancer	PIK3CA	p.Glu545Lys	3_178936091_G_A	0.08443323	TRUE	FALSE	FALSE	TRUE	TRUE
Colorectal Cancer	FBXW7	p.Arg505Cys	4_153247289_G_A	0.003846154	FALSE	FALSE	FALSE	TRUE	FALSE
Colorectal Cancer	APC	p.Glu991*	5_112174262_G_T	0.034469097	TRUE	FALSE	FALSE	TRUE	TRUE
Colorectal Cancer	APC	p.Gln1444*	5_112175621_C_T	0.080998563	TRUE	FALSE	FALSE	TRUE	TRUE

Non-Small Cell Lung Cancer	TP53	p.Trp91*	17_7579414_C_T	0.003558719	TRUE	FALSE	FALSE	TRUE	TRUE
Non-Small Cell Lung Cancer	EGFR	p.Glu746_Ala750 del	7_55242464_AGGAATT AAGAGAAGC_A	0.022773902	TRUE	FALSE	FALSE	TRUE	TRUE
Breast Cancer	TP53	p.Val272Leu	17_7577124_C_A	0.002840909	FALSE	FALSE	FALSE	TRUE	FALSE
Breast Cancer	TP53	p.Arg213*	17_7578212_G_A	0.035663338	TRUE	FALSE	FALSE	TRUE	TRUE
Breast Cancer	ERBB2	p.Asp1252Glu	17_37884285_C_G	0.014128728	TRUE	FALSE	FALSE	TRUE	TRUE
Breast Cancer	BRCA1	p.Glu809Lys	17_41245123_C_T	0.0155521	TRUE	FALSE	FALSE	TRUE	TRUE
Breast Cancer	MSH6	p.Glu30Lys	2_48010460_G_A	0.005681818	FALSE	FALSE	FALSE	TRUE	FALSE
Breast Cancer	PIK3CA	p.Gly106_Arg108 del	3_178916927_TAGGCA ACCG_T	0.015990862	TRUE	FALSE	FALSE	TRUE	TRUE
Breast Cancer	TP53	p.Tyr220Cys	17_7578190_T_C	0.036917194	TRUE	FALSE	FALSE	TRUE	TRUE
Breast Cancer	DDR2	p.Arg680Gly	1_162745623_C_G	0.584649177	FALSE	TRUE	FALSE	TRUE	TRUE
Breast Cancer	TP53	p.Gln165*	17_7578437_G_A	0.320960699	TRUE	TRUE	TRUE	TRUE	TRUE
Breast Cancer	MYCN	p.Ala354Val	2_16085885_C_T	0.543351232	FALSE	TRUE	FALSE	TRUE	TRUE
Breast Cancer	ATR	p.His2437Tyr	3_142178109_G_A	0.034273474	FALSE	FALSE	FALSE	TRUE	FALSE
Breast Cancer	FGFR3	0	4_1809420_C_A	0.711139659	FALSE	TRUE	FALSE	TRUE	TRUE
Breast Cancer	ATM	p.Tyr1470His	11_108160500_T_C	0.003415301	FALSE	FALSE	FALSE	TRUE	FALSE
Breast Cancer	FGFR3	0	4_1809841_A_G	0.005329273	FALSE	TRUE	TRUE	TRUE	TRUE
Colorectal Cancer	KRAS	p.Gly12Cys	12_25398285_C_A	0.117774678	TRUE	FALSE	FALSE	TRUE	TRUE
Colorectal Cancer	APC	p.Tyr1031*	5_112174384_T_G	0.038877481	TRUE	FALSE	FALSE	TRUE	TRUE
Colorectal Cancer	APC	p.Arg1450*	5_112175639_C_T	0.042755079	TRUE	FALSE	FALSE	TRUE	TRUE
Colorectal Cancer	KRAS	p.Gly12Val	12_25398284_C_A	0.537948084	TRUE	TRUE	TRUE	TRUE	TRUE
Colorectal Cancer	TSC2	p.Thr831Met	16_2124337_C_T	0.422265665	TRUE	FALSE	FALSE	TRUE	TRUE
Colorectal Cancer	APC	p.Ser1346*	5_112175328_C_A	0.597274371	TRUE	TRUE	TRUE	TRUE	TRUE
Colorectal Cancer	BRCA2	p.Gly1376Ala	13_32912619_G_C	0.002997602	FALSE	FALSE	FALSE	TRUE	FALSE
Colorectal Cancer	TP53	p.Arg248Trp	17_7577539_G_A	0.114472863	TRUE	FALSE	FALSE	TRUE	TRUE
Colorectal Cancer	BRCA1	p.Pro1502Leu	17_41226518_G_A	0.172706082	TRUE	TRUE	TRUE	TRUE	TRUE
Colorectal Cancer	APC	p.Arg232*	5_112128191_C_T	0.101369863	TRUE	FALSE	FALSE	TRUE	TRUE
Colorectal Cancer	NRAS	p.Ala146Thr	1_115252204_C_T	0.783776327	TRUE	FALSE	FALSE	TRUE	TRUE
Colorectal Cancer	TP53	p.Gly262del	17_7577151_TACC_T	0.755041955	TRUE	FALSE	FALSE	TRUE	TRUE
Colorectal Cancer	APC	p.Thr1023fs	5_112174356_A_AT	0.227348643	TRUE	TRUE	TRUE	TRUE	TRUE
Colorectal Cancer	APC	p.Gln1429*	5_112175576_C_T	0.534669846	TRUE	FALSE	FALSE	TRUE	TRUE
Colorectal Cancer	BRAF	p.Asp594Gly	7_140453154_T_C	0.523831996	TRUE	FALSE	FALSE	TRUE	TRUE
Colorectal Cancer	MYC	p.Asn323Lys	8_128752808_C_G	0.003378815	FALSE	FALSE	FALSE	TRUE	FALSE
Breast Cancer	TP53	p.Arg248Gln	17_7577538_C_T	0.003671329	FALSE	FALSE	FALSE	TRUE	FALSE
Breast Cancer	TP53	p.Arg110fs	17_7579363_A_AC	0.015791119	TRUE	FALSE	FALSE	TRUE	TRUE
Breast Cancer	MAP2K2	p.Val283Met	19_4099271_C_T	0.448767834	FALSE	TRUE	FALSE	TRUE	TRUE
Breast Cancer	CDKN2A	p.Ser12Leu	9_21974792_G_A	0.003799392	FALSE	FALSE	FALSE	TRUE	FALSE
Breast Cancer	AR	p.Met788Val	X_66941718_A_G	0.003796507	FALSE	FALSE	FALSE	TRUE	FALSE

Breast Cancer	ARID1A	p.Glu1542Lys	1_27101342_G_A	0.006855041	FALSE	FALSE	FALSE	TRUE	FALSE
Breast Cancer	ARID1A	p.Glu1786Gln	1_27105745_G_C	0.008781783	FALSE	FALSE	FALSE	TRUE	FALSE
Breast Cancer	HRAS	p.Lys117Asn	11_533552_C_A	0.024554	FALSE	FALSE	FALSE	TRUE	FALSE
Breast Cancer	HRAS	p.Gln61Leu	11_533874_T_A	0.015660416	FALSE	FALSE	FALSE	TRUE	FALSE
Breast Cancer	HRAS	p.Gln61Lys	11_533875_G_T	0.007129762	TRUE	FALSE	FALSE	TRUE	TRUE
Breast Cancer	HRAS	p.Gly13Arg	11_534286_C_G	0.006965729	FALSE	FALSE	FALSE	TRUE	FALSE
Breast Cancer	HRAS	p.Gly12Asp	11_534288_C_T	0.024500141	FALSE	FALSE	FALSE	TRUE	FALSE
Breast Cancer	HRAS	p.Gly12Ser	11_534289_C_T	0.00737798	FALSE	FALSE	FALSE	TRUE	FALSE
Breast Cancer	ATM	p.Glu359Lys	11_108119669_G_A	0.049607753	FALSE	FALSE	FALSE	TRUE	FALSE
Breast Cancer	ATM	p.Glu522Lys	11_108121756_G_A	0.003866122	FALSE	FALSE	FALSE	TRUE	FALSE
Breast Cancer	ATM	p.Glu2402Lys	11_108199862_G_A	0.006512974	FALSE	FALSE	FALSE	TRUE	FALSE
Breast Cancer	KMT2A	p.Ser779Leu	11_118344210_C_T	0.005580493	FALSE	FALSE	FALSE	TRUE	FALSE
Breast Cancer	KMT2A	p.Ser996Cys	11_118344861_C_G	0.005039194	FALSE	FALSE	FALSE	TRUE	FALSE
Breast Cancer	KMT2A	p.Asp2039Asn	11_118370585_G_A	0.004664824	FALSE	FALSE	FALSE	TRUE	FALSE
Breast Cancer	CDK4	p.Pro302Ala	12_58142316_G_C	0.005274115	FALSE	FALSE	FALSE	TRUE	FALSE
Breast Cancer	PTPN11	p.Ser109Cys	12_112888310_C_G	0.042517388	FALSE	FALSE	FALSE	TRUE	FALSE
Breast Cancer	BRCA2	p.Arg3269Thr	13_32972456_G_C	0.004942107	FALSE	FALSE	FALSE	TRUE	FALSE
Breast Cancer	AKT1	p.Ser381Leu	14_105239245_G_A	0.079777605	FALSE	FALSE	FALSE	TRUE	FALSE
Breast Cancer	TSC2	p.Glu1490Lys	16_2134691_G_A	0.006543321	FALSE	FALSE	FALSE	TRUE	FALSE
Breast Cancer	TSC2	p.Asp1613Asn	16_2136368_G_A	0.003895755	FALSE	FALSE	FALSE	TRUE	FALSE
Breast Cancer	CDH1	p.Asp402Asn	16_68847282_G_A	0.003702457	FALSE	FALSE	FALSE	TRUE	FALSE
Breast Cancer	NF1	p.Arg461*	17_29533378_C_T	0.010514541	FALSE	FALSE	FALSE	TRUE	FALSE
Breast Cancer	NF1	p.Gln535*	17_29546098_C_T	0.003166436	FALSE	FALSE	FALSE	TRUE	FALSE
Breast Cancer	NF1	p.Glu595*	17_29550523_G_T	0.008386103	FALSE	FALSE	FALSE	TRUE	FALSE
Breast Cancer	NF1	p.Glu648*	17_29552209_G_T	0.00950951	FALSE	FALSE	FALSE	TRUE	FALSE
Breast Cancer	NF1	p.Ser879*	17_29556269_C_A	0.009746013	FALSE	FALSE	FALSE	TRUE	FALSE
Breast Cancer	NF1	p.Gln1017*	17_29557336_C_T	0.005352364	FALSE	FALSE	FALSE	TRUE	FALSE
Breast Cancer	NF1	p.Glu1089*	17_29559158_G_T	0.002709293	FALSE	FALSE	FALSE	TRUE	FALSE
Breast Cancer	NF1	p.Ser2549*	17_29683508_C_G	0.020675744	FALSE	FALSE	FALSE	TRUE	FALSE
Breast Cancer	ERBB2	p.Ile767Met	17_37880257_C_G	0.009242403	FALSE	FALSE	FALSE	TRUE	FALSE
Breast Cancer	ERBB2	p.Phe1213Leu	17_37884168_C_G	0.035407979	FALSE	FALSE	FALSE	TRUE	FALSE
Breast Cancer	STK11	p.Gln100Glu	19_1218423_C_G	0.002585551	FALSE	FALSE	FALSE	TRUE	FALSE
Breast Cancer	KEAP1	p.Ter625Ter	19_10597329_C_T	0.003249151	FALSE	FALSE	FALSE	TRUE	FALSE
Breast Cancer	MYCN	p.Glu132Asp	2_16082582_G_C	0.011609158	FALSE	FALSE	FALSE	TRUE	FALSE
Breast Cancer	MSH2	p.Gln337Glu	2_47643501_C_G	0.004267425	FALSE	FALSE	FALSE	TRUE	FALSE
Breast Cancer	MSH6	p.Glu983Gln	2_48028069_G_C	0.006472903	FALSE	FALSE	FALSE	TRUE	FALSE
Breast Cancer	MSH6	p.Gln1258Lys	2_48033468_C_A	0.003982477	FALSE	FALSE	FALSE	TRUE	FALSE
Breast Cancer	MAPK1	p.Ile53Met	22_22162096_G_C	0.002728732	FALSE	FALSE	FALSE	TRUE	FALSE

Breast Cancer	MLH1	p.Gly174Arg	3_37050371_G_A	0.003163723	FALSE	FALSE	FALSE	TRUE	FALSE
Breast Cancer	MLH1	p.Glu199Lys	3_37053508_G_A	0.005426221	FALSE	FALSE	FALSE	TRUE	FALSE
Breast Cancer	PBRM1	p.Glu602Lys	3_52651292_C_T	0.011056705	FALSE	FALSE	FALSE	TRUE	FALSE
Breast Cancer	ATR	p.Glu1050Gln	3_142268344_C_G	0.016447368	FALSE	FALSE	FALSE	TRUE	FALSE
Breast Cancer	PIK3CA	p.Arg93Trp	3_178916890_C_T	0.083247399	TRUE	FALSE	FALSE	TRUE	TRUE
Breast Cancer	PIK3CA	p.Glu542Lys	3_178936082_G_A	0.641898031	TRUE	TRUE	TRUE	TRUE	TRUE
Breast Cancer	PIK3CA	p.Met1043Ile	3_178952074_G_C	0.005177869	FALSE	FALSE	FALSE	TRUE	FALSE
Breast Cancer	PDGFRB	p.Met743Ile	5_149501558_C_T	0.209450964	TRUE	TRUE	TRUE	TRUE	TRUE
Breast Cancer	NPM1	0	5_170814952_G_A	0.005417118	FALSE	FALSE	FALSE	TRUE	FALSE
Breast Cancer	CDK6	p.Glu263Gln	7_92247433_C_G	0.00306777	FALSE	FALSE	FALSE	TRUE	FALSE
Breast Cancer	MET	p.Arg331Thr	7_116340130_G_C	0.06072322	FALSE	FALSE	FALSE	TRUE	FALSE
Breast Cancer	BRAF	p.Ser579Cys	7_140453993_T_A	0.122423572	TRUE	FALSE	FALSE	TRUE	TRUE
Breast Cancer	FGFR1	p.Glu41Lys	8_38287437_C_T	0.004782973	FALSE	FALSE	FALSE	TRUE	FALSE
Breast Cancer	JAK2	p.Arg300Thr	9_5054847_G_C	0.005012531	FALSE	FALSE	FALSE	TRUE	FALSE
Breast Cancer	PTCH1	p.Gln343His	9_98240457_C_G	0.004846527	FALSE	FALSE	FALSE	TRUE	FALSE
Breast Cancer	TSC1	p.Gly999Glu	9_135772121_C_T	0.005365526	FALSE	FALSE	FALSE	TRUE	FALSE
Breast Cancer	TSC1	p.Ala726Pro	9_135779070_C_G	0.343185706	TRUE	FALSE	FALSE	TRUE	TRUE
Breast Cancer	TSC1	p.His279Asn	9_135787747_G_T	0.051254089	FALSE	FALSE	FALSE	TRUE	FALSE
Breast Cancer	AR	p.Ser754Phe	X_66937407_C_T	0.065795304	FALSE	FALSE	FALSE	TRUE	FALSE
Colorectal Cancer	ERRFI1	p.Arg199Gln	1_8074063_C_T	0.077787022	TRUE	FALSE	FALSE	TRUE	TRUE
Colorectal Cancer	KRAS	p.Gly12Asp	12_25398284_C_T	0.088731628	TRUE	FALSE	FALSE	TRUE	TRUE
Colorectal Cancer	PIK3CA	p.Arg88Gln	3_178916876_G_A	0.092076302	TRUE	TRUE	TRUE	TRUE	TRUE
Colorectal Cancer	APC	p.Arg232*	5_112128191_C_T	0.048142334	TRUE	FALSE	FALSE	TRUE	TRUE
Breast Cancer	TP53	p.Phe270Ser	17_7577129_A_G	0.005781866	FALSE	FALSE	FALSE	TRUE	FALSE
Breast Cancer	NF2	p.Arg424His	22_30069406_G_A	0.493509674	FALSE	TRUE	FALSE	TRUE	TRUE
Non-Small Cell Lung Cancer	TP53	p.His178_Glu180 del	17_7578388_CGCTCAT GGT_C	0.016820113	TRUE	FALSE	FALSE	TRUE	TRUE
Non-Small Cell Lung Cancer	ALK	p.Ser1034Trp	2_29448398_G_C	0.016483516	TRUE	FALSE	FALSE	TRUE	TRUE
Non-Small Cell Lung Cancer	EGFR	p.Pro772_His773 dup	7_55249013_A_AACCC CC	0.018570427	TRUE	FALSE	FALSE	TRUE	TRUE
Colorectal Cancer	KRAS	p.Gly13Asp	12_25398281_C_T	0.309334545	TRUE	FALSE	FALSE	TRUE	TRUE
Colorectal Cancer	TP53	p.Arg282fs	17_7577093_CG_C	0.246124443	TRUE	FALSE	FALSE	TRUE	TRUE
Colorectal Cancer	APC	p.Glu1020*	5_112174349_G_T	0.137354651	TRUE	TRUE	TRUE	TRUE	TRUE
Colorectal Cancer	TSC1	p.His253Pro	9_135787824_T_G	0.002744425	FALSE	FALSE	FALSE	TRUE	FALSE
Colorectal Cancer	EGFR	p.Ser991Gly	7_55268905_A_G	0.002568807	FALSE	TRUE	TRUE	TRUE	TRUE
Colorectal Cancer	TP53	p.Arg175His	17_7578406_C_T	0.285656647	TRUE	FALSE	FALSE	TRUE	TRUE
Colorectal Cancer	FBXW7	p.Asp101Asn	4_153332655_C_T	0.543636909	FALSE	TRUE	FALSE	TRUE	TRUE
Colorectal Cancer	APC	p.Leu1302fs	5_112175192_AC_A	0.338499338	TRUE	FALSE	FALSE	TRUE	TRUE
Colorectal Cancer	KRAS	p.Gly13Asp	12_25398281_C_T	0.006015733	FALSE	FALSE	FALSE	TRUE	FALSE

Colorectal Cancer	CDH1	p.Gly760Ala	16_68862191_G_C	0.002991027	FALSE	FALSE	FALSE	TRUE	FALSE
Colorectal Cancer	TP53	p.Arg342*	17_7574003_G_A	0.214224064	TRUE	TRUE	TRUE	TRUE	TRUE
Colorectal Cancer	RNF43	p.Ter784Glyext*?	17_56432306_A_C	0.002539503	FALSE	FALSE	FALSE	TRUE	FALSE
Colorectal Cancer	APC	p.Arg232*	5_112128191_C_T	0.132352941	TRUE	FALSE	FALSE	TRUE	TRUE
Colorectal Cancer	APC	p.Lys1363*	5_112175378_A_T	0.261152594	TRUE	FALSE	FALSE	TRUE	TRUE
Colorectal Cancer	TP53	p.Tyr220Ser	17_7578190_T_G	0.003082456	FALSE	FALSE	FALSE	TRUE	FALSE
Colorectal Cancer	PIK3R1	p.Ser652fs	5_67592105_A_AAGCG AGATGGCACTTTTCT TGTCCGGGAG	0.009954243	FALSE	FALSE	FALSE	TRUE	FALSE
Colorectal Cancer	FGFR2	p.Lys310Gln	10_123279504_T_G	0.002779884	FALSE	FALSE	FALSE	TRUE	FALSE
Colorectal Cancer	KMT2A	p.Thr2089Asn	11_118371809_C_A	0.536011629	FALSE	TRUE	FALSE	TRUE	TRUE
Colorectal Cancer	KRAS	p.Gly12Val	12_25398284_C_A	0.106908222	TRUE	FALSE	FALSE	TRUE	TRUE
Colorectal Cancer	TP53	p.Arg175His	17_7578406_C_T	0.183235541	TRUE	FALSE	FALSE	TRUE	TRUE
Colorectal Cancer	ALK	p.Ile938Thr	2_29451752_A_G	0.522542978	FALSE	TRUE	FALSE	TRUE	TRUE
Colorectal Cancer	ALK	p.Gly872Ser	2_29455188_C_T	0.069724284	TRUE	FALSE	FALSE	TRUE	TRUE
Colorectal Cancer	CTNNB1	p.Leu262Arg	3_41267201_T_G	0.074478178	TRUE	TRUE	TRUE	TRUE	TRUE
Colorectal Cancer	APC	p.Gln1303*	5_112175198_C_T	0.072367298	TRUE	FALSE	FALSE	TRUE	TRUE
Colorectal Cancer	NTRK1	p.Arg554Gln	1_156846220_G_A	0.510586539	FALSE	TRUE	FALSE	TRUE	TRUE
Colorectal Cancer	GATA3	p.Val440Phe	10_8115969_G_T	0.463465553	FALSE	TRUE	FALSE	TRUE	TRUE
Colorectal Cancer	TP53	p.Gly245Ser	17_7577548_C_T	0.071586173	TRUE	FALSE	FALSE	TRUE	TRUE
Colorectal Cancer	NFE2L2	p.Ala72Thr	2_178098831_C_T	0.515858834	FALSE	TRUE	FALSE	TRUE	TRUE
Colorectal Cancer	MSH3	p.Ala55_Ala57dup	5_79950696_C_CGCTG CAGCG	0.377949853	FALSE	TRUE	TRUE	TRUE	TRUE
Colorectal Cancer	APC	p.Glu1309fs	5_112175211_TAAAAG T	0.109776833	TRUE	FALSE	FALSE	TRUE	TRUE
Breast Cancer	TP53	p.Arg273Cys	17_7577121_G_A	0.003145753	FALSE	FALSE	FALSE	TRUE	FALSE
Colorectal Cancer	KRAS	p.Gly12Val	12_25398284_C_A	0.055505979	TRUE	FALSE	FALSE	TRUE	TRUE
Colorectal Cancer	SMAD4	p.Ala118Val	18_48575159_C_T	0.074473925	TRUE	FALSE	FALSE	TRUE	TRUE
Colorectal Cancer	PDGFRA	p.Leu47Arg	4_55127352_T_G	0.005228758	FALSE	FALSE	FALSE	TRUE	FALSE
Colorectal Cancer	APC	p.Ser748fs	5_112173531_CAAGCT TGCCATCTCTTCATG TTAGGAAACAA_C	0.042661393	TRUE	FALSE	FALSE	TRUE	TRUE
Colorectal Cancer	APC	p.Gln1429*	5_112175576_C_T	0.073447063	TRUE	FALSE	FALSE	TRUE	TRUE
Colorectal Cancer	NOTCH1	p.Ala1741Val	9_139396886_G_A	0.092554819	TRUE	TRUE	TRUE	TRUE	TRUE
Breast Cancer	TP53	p.Arg213*	17_7578212_G_A	0.154423381	TRUE	TRUE	TRUE	TRUE	TRUE
Breast Cancer	SDHA	p.Gln176His	5_226069_G_C	0.004660453	FALSE	TRUE	TRUE	TRUE	TRUE
Colorectal Cancer	PTEN	p.Tyr27Cys	10_89653782_A_G	0.555891239	TRUE	FALSE	FALSE	FALSE	FALSE
Colorectal Cancer	KRAS	p.Gly12Val	12_25398284_C_A	0.325524044	TRUE	FALSE	FALSE	FALSE	FALSE

Colorectal Cancer	PIK3CA	p.Met1043Val	3_178952072_A_G	0.329820864	TRUE	FALSE	FALSE	FALSE	FALSE
Breast Cancer	ARID1A	p.Gln1584*	1_27101468_C_T	0.453908985	TRUE	FALSE	FALSE	FALSE	FALSE
Colorectal Cancer	KRAS	p.Gly12Asp	12_25398284_C_T	0.152322404	TRUE	FALSE	FALSE	FALSE	FALSE
Colorectal Cancer	CTNNB1	p.Cys213Phe	3_41266967_G_T	0.153005464	TRUE	FALSE	FALSE	FALSE	FALSE
Colorectal Cancer	APC	p.Asn1531fs	5_112175879_GA_G	0.300892133	TRUE	FALSE	FALSE	FALSE	FALSE
Colorectal Cancer	PTCH1	p.Thr1304Ile	9_98209429_G_A	0.142448103	TRUE	FALSE	FALSE	FALSE	FALSE
Colorectal Cancer	KRAS	p.Gly12Val	12_25398284_C_A	0.284156977	TRUE	FALSE	FALSE	FALSE	FALSE
Colorectal Cancer	TP53	p.Glu287fs	17_7577079_CT_C	0.447183099	TRUE	FALSE	FALSE	FALSE	FALSE
Colorectal Cancer	APC	p.Arg805*	5_112173704_C_T	0.291484716	TRUE	FALSE	FALSE	FALSE	FALSE
Non-Small Cell Lung Cancer	TP53	p.Gly154fs	17_7578469_CCG_AAC T	0.324324324	TRUE	FALSE	FALSE	FALSE	FALSE
Non-Small Cell Lung Cancer	RET	p.Val347Leu	10_43601995_G_T	0.341614907	TRUE	FALSE	FALSE	FALSE	FALSE
Non-Small Cell Lung Cancer	FGFR2	p.Gly485Cys	10_123260451_C_A	0.236421725	TRUE	FALSE	FALSE	FALSE	FALSE
Non-Small Cell Lung Cancer	TP53	p.Gln136fs	17_7578523_TG_T	0.369649805	TRUE	FALSE	FALSE	FALSE	FALSE
Non-Small Cell Lung Cancer	ESR1	p.Glu339Asp	6_152265564_A_T	0.331658291	TRUE	FALSE	FALSE	FALSE	FALSE
Non-Small Cell Lung Cancer	NOTCH1	p.Pro407Leu	9_139412623_CG_TA	0.738095238	TRUE	FALSE	FALSE	FALSE	FALSE
Breast Cancer	JAK1	p.Ser909Cys	1_65305402_G_C	0.051643192	TRUE	FALSE	FALSE	FALSE	FALSE
Breast Cancer	RB1	p.Arg698Gly	13_49033955_A_G	0.392523364	TRUE	FALSE	FALSE	FALSE	FALSE
Breast Cancer	TP53	p.Glu285Lys	17_7577085_C_T	0.31372549	TRUE	FALSE	FALSE	FALSE	FALSE
Breast Cancer	TP53	p.Arg282Trp	17_7577094_G_A	0.662337662	TRUE	FALSE	FALSE	FALSE	FALSE
Breast Cancer	PIK3CA	p.His1047Arg	3_178952085_A_G	0.789473684	TRUE	FALSE	FALSE	FALSE	FALSE
Breast Cancer	PDGFRB	p.Glu390Lys	5_149511617_C_T	0.232323232	TRUE	FALSE	FALSE	FALSE	FALSE
Breast Cancer	CD274	p.Phe191Leu	9_5463012_C_G	0.164874552	TRUE	FALSE	FALSE	FALSE	FALSE
Non-Small Cell Lung Cancer	ARID1A	p.Ser138*	1_27023307_C_G	0.21686747	TRUE	FALSE	FALSE	FALSE	FALSE
Non-Small Cell Lung Cancer	BRCA2	p.Ser193Cys	13_32900697_C_G	0.234693878	TRUE	FALSE	FALSE	FALSE	FALSE
Breast Cancer	PIK3CA	p.His1047Arg	3_178952085_A_G	0.316384181	TRUE	FALSE	FALSE	FALSE	FALSE
Breast Cancer	TERT		5_1295228_G_A	0.32972973	TRUE	FALSE	FALSE	FALSE	FALSE
Colorectal Cancer	EGFR	p.Ala16Thr	7_55087016_G_A	0.060176991	TRUE	FALSE	FALSE	FALSE	FALSE
Breast Cancer	RB1	p.Ser178Arg	13_48921994_C_G	0.224358974	TRUE	FALSE	FALSE	FALSE	FALSE
Breast Cancer	MYCN	p.Glu378Lys	2_16085956_G_A	0.214244016	TRUE	FALSE	FALSE	FALSE	FALSE
Breast Cancer	MET	p.Asp243His	7_116339865_G_C	0.210175651	TRUE	FALSE	FALSE	FALSE	FALSE
Colorectal Cancer	KRAS	p.Gly12Asp	12_25398284_C_T	0.29460896	TRUE	FALSE	FALSE	FALSE	FALSE
Colorectal Cancer	ERBB2	p.Ser358Arg	17_37868627_T_A	0.331533477	TRUE	FALSE	FALSE	FALSE	FALSE
Colorectal Cancer	SMAD4	p.Gln284*	18_48584772_C_T	0.444370861	TRUE	FALSE	FALSE	FALSE	FALSE
Colorectal Cancer	ALK	p.Gly394Arg	2_29606700_C_T	0.289368505	TRUE	FALSE	FALSE	FALSE	FALSE
Colorectal Cancer	APC	p.Arg499*	5_112162891_C_T	0.274414063	TRUE	FALSE	FALSE	FALSE	FALSE
Colorectal Cancer	APC	p.Asn1455fs	5_112175649_CT_C	0.317272727	TRUE	FALSE	FALSE	FALSE	FALSE
Colorectal Cancer	ATM	p.Gln1015Lys	11_108142099_C_A	0.051546392	TRUE	FALSE	FALSE	FALSE	FALSE

Colorectal Cancer	HNF1A	p.Pro507Ser	12_121437088_C_T	0.059405941	TRUE	FALSE	FALSE	FALSE	FALSE
Colorectal Cancer	AKT1	p.Arg25His	14_105246526_C_T	0.08	TRUE	FALSE	FALSE	FALSE	FALSE
Colorectal Cancer	FGFR3	p.Thr171Ile	4_1803160_C_T	0.055045872	TRUE	FALSE	FALSE	FALSE	FALSE
Colorectal Cancer	FGFR3	p.Pro573Ser	4_1807548_C_T	0.05	TRUE	FALSE	FALSE	FALSE	FALSE
Colorectal Cancer	KIT	p.Pro944Leu	4_55604623_C_T	0.053333333	TRUE	FALSE	FALSE	FALSE	FALSE
Colorectal Cancer	KDR	p.Glu759Lys	4_55964962_C_T	0.08	TRUE	FALSE	FALSE	FALSE	FALSE
Colorectal Cancer	TERT		5_1295123_C_T	0.05	TRUE	FALSE	FALSE	FALSE	FALSE
Colorectal Cancer	PIK3R1	p.Val695Met	5_67593337_G_A	0.058823529	TRUE	FALSE	FALSE	FALSE	FALSE
Colorectal Cancer	APC	p.Arg332*	5_112154723_C_T	0.058823529	TRUE	FALSE	FALSE	FALSE	FALSE
Colorectal Cancer	GNAQ	p.Ala93Val	9_80537120_G_A	0.050632911	TRUE	FALSE	FALSE	FALSE	FALSE
Colorectal Cancer	TSC1	p.Arg204His	9_135797258_C_T	0.051282051	TRUE	FALSE	FALSE	FALSE	FALSE
Colorectal Cancer	TERT	p.Gln40*	5_1294987_G_A	0.054054054	TRUE	FALSE	FALSE	FALSE	FALSE
Colorectal Cancer	TP53	p.Arg175His	17_7578406_C_T	0.475452196	TRUE	FALSE	FALSE	FALSE	FALSE
Colorectal Cancer	ERBB2	p.Ser310Tyr	17_37868208_C_A	0.332159624	TRUE	FALSE	FALSE	FALSE	FALSE
Breast Cancer	TP53	p.Arg175His	17_7578406_C_T	0.817460317	TRUE	FALSE	FALSE	FALSE	FALSE
Breast Cancer	BRCA2	p.Glu2846Gln	13_32945141_G_C	0.296875	TRUE	FALSE	FALSE	FALSE	FALSE
Breast Cancer	SMAD4	p.Cys127fs	18_48575184_CTGTG_C	0.433884298	TRUE	FALSE	FALSE	FALSE	FALSE
Breast Cancer	PIK3CA	p.His1047Arg	3_178952085_A_G	0.300163132	TRUE	FALSE	FALSE	FALSE	FALSE
Non-Small Cell Lung Cancer	ARID1A	p.Met1564fs	1_27101408_A_CT	0.191873589	TRUE	FALSE	FALSE	FALSE	FALSE
Non-Small Cell Lung Cancer	RET	p.Pro992Thr	10_43620365_C_A	0.391442155	TRUE	FALSE	FALSE	FALSE	FALSE
Non-Small Cell Lung Cancer	TP53	p.Ser215Ile	17_7578205_C_A	0.171161826	TRUE	FALSE	FALSE	FALSE	FALSE
Non-Small Cell Lung Cancer	TP53	p.Glu68*	17_7579485_C_A	0.550680787	TRUE	FALSE	FALSE	FALSE	FALSE
Non-Small Cell Lung Cancer	STK11	p.Arg310fs	19_1222989_T_TC	0.583832335	TRUE	FALSE	FALSE	FALSE	FALSE
Non-Small Cell Lung Cancer	EGFR	p.Val765Met	7_55248995_G_A	0.152066116	TRUE	FALSE	FALSE	FALSE	FALSE
Non-Small Cell Lung Cancer	AR	p.Gly418Cys	X_66766240_G_T	0.585365854	TRUE	FALSE	FALSE	FALSE	FALSE
Colorectal Cancer	KRAS	p.Gly13Asp	12_25398281_C_T	0.412875536	TRUE	FALSE	FALSE	FALSE	FALSE
Colorectal Cancer	FBXW7	p.Arg465Cys	4_153249385_G_A	0.174208145	TRUE	FALSE	FALSE	FALSE	FALSE
Colorectal Cancer	APC	p.Gln1429*	5_112175576_C_T	0.571763327	TRUE	FALSE	FALSE	FALSE	FALSE
Colorectal Cancer	CDKN2A	p.Asp74Asn	9_21971138_C_T	0.29890644	TRUE	FALSE	FALSE	FALSE	FALSE
Non-Small Cell Lung Cancer	TP53	p.Val157Phe	17_7578461_C_A	0.220472441	TRUE	FALSE	FALSE	FALSE	FALSE
Non-Small Cell Lung Cancer	TP53	p.Ala83fs	17_7579439_G_GC	0.400452489	TRUE	FALSE	FALSE	FALSE	FALSE
Non-Small Cell Lung Cancer	SMO	p.Pro13Ser	7_128829029_C_T	0.268292683	TRUE	FALSE	FALSE	FALSE	FALSE
Breast Cancer	ATR	p.Thr2180Ile	3_142188192_G_A	0.066825776	TRUE	FALSE	FALSE	FALSE	FALSE
Breast Cancer	ARID1A	p.Gly108fs	1_27023212_CGCGGGCCCTAGGCCCGCCCTGAACAATAACCTCACGGAGCCGCCCGGCG_C	0.234513274	TRUE	FALSE	FALSE	FALSE	FALSE

Colorectal Cancer	ERBB2	p.Arg47Cys	17_37863308_C_T	0.11281337	TRUE	FALSE	FALSE	FALSE	FALSE
Colorectal Cancer	MSH6	p.Phe1088fs	2_48030639_A_AC	0.127320955	TRUE	FALSE	FALSE	FALSE	FALSE
Colorectal Cancer	MET		7_116436186_A_G	0.05794702	TRUE	FALSE	FALSE	FALSE	FALSE
Colorectal Cancer	STK11	p.Pro339Thr	19_1223078_C_A	0.051546392	TRUE	FALSE	FALSE	FALSE	FALSE

Supplementary Table 5. Cancer groups included in xF 1000 clinical profiling analysis

Cancer Group	Count
Breast	254
Lung	241
Colorectal	98
Prostate	96
Pancreatic	83
Tumor of unknown origin	28
Hepatobiliary	26
Ovarian	23
Melanoma	21
Head and neck	19
Bladder	18
Gastric	18
Kidney	18
Sarcoma	14
Esophageal	10
Endometrial	8
Thyroid	6
Endocrine	6
Skin	4
Thymoma	3
Cervical	2
Testicular	2
Glioma	1
Mesothelioma	1
